# Rapid and direct control of target protein levels with VHL-recruiting dTAG molecules

**DOI:** 10.1038/s41467-020-18377-w

**Published:** 2020-09-18

**Authors:** Behnam Nabet, Fleur M. Ferguson, Bo Kyung A. Seong, Miljan Kuljanin, Alan L. Leggett, Mikaela L. Mohardt, Amanda Robichaud, Amy S. Conway, Dennis L. Buckley, Joseph D. Mancias, James E. Bradner, Kimberly Stegmaier, Nathanael S. Gray

**Affiliations:** 1 Department of Cancer Biology, Dana-Farber Cancer Institute, Boston, MA USA; 2 Department of Biological Chemistry and Molecular Pharmacology, Harvard Medical School, Boston, MA USA; 3 Department of Pediatric Oncology, Dana-Farber Cancer Institute, Boston, MA USA; 4 The Broad Institute of MIT and Harvard, Cambridge, MA USA; 5 Division of Radiation and Genome Stability, Department of Radiation Oncology, Dana-Farber Cancer Institute, Boston, MA USA; 6 Department of Medical Oncology, Dana-Farber Cancer Institute, Boston, MA USA; 7 Department of Medicine, Harvard Medical School, Boston, MA USA; 8 Division of Pediatric Hematology/Oncology, Boston Children’s Hospital, Boston, MA USA; 9 Present Address: Novartis Institutes for BioMedical Research, Cambridge, MA USA

**Keywords:** Proteolysis, Chemical tools, Target validation

## Abstract

Chemical biology strategies for directly perturbing protein homeostasis including the degradation tag (dTAG) system provide temporal advantages over genetic approaches and improved selectivity over small molecule inhibitors. We describe dTAG^V^-1, an exclusively selective VHL-recruiting dTAG molecule, to rapidly degrade FKBP12^F36V^-tagged proteins. dTAG^V^-1 overcomes a limitation of previously reported CRBN-recruiting dTAG molecules to degrade recalcitrant oncogenes, supports combination degrader studies and facilitates investigations of protein function in cells and mice.

## Abstract (web summary)

The dTAG system is used to rapidly deplete tagged target proteins in vitro and in vivo, but there are context- and protein-specific differences in its effectiveness. Here, the authors develop a second generation dTAG molecule that can degrade previously recalcitrant target proteins in cells and mice.

## Introduction

Modulating protein abundance with small molecule degraders is a powerful approach for investigating functional consequences of rapid and direct protein loss, without alteration of corresponding mRNA levels. Degraders including heterobifunctional degraders (also known as PROteolysis-TArgeting Chimeras or PROTACs) and nonchimeric molecular glues, co-opt an E3 ubiquitin ligase to induce rapid and reversible proteasome-mediated degradation^1^. Achieving immediate target protein loss with degraders provides a crucial advantage over genetic knockout or knockdown approaches, which require a significant delay to achieve an impactful protein reduction^2^. However, degrader development is hindered by a reliance on target-specific chemical matter, which is unavailable for the majority of the proteome. To address this challenge, several strategies aimed at the direct control of cellular protein levels have been recently developed, including methods that use small molecules^3–9^, nanobodies^10^, or antibodies^11^.

We previously described a versatile approach known as the degradation tag (dTAG) system to rapidly deplete any tagged target protein in cells and in mice^6^. The dTAG system is a dual component platform requiring the expression of FKBP12^F36V^ in-frame with a gene-of-interest and treatment with a heterobifunctional dTAG molecule (dTAG-13)^12^ that engages FKBP12^F36V^ and cereblon (CRBN), an E3 ubiquitin ligase (Supplementary Fig. 1a, b). This interaction leads to exclusive degradation of the FKBP12^F36V^-tagged protein. Studies degrading diverse targets including oncoproteins, transcription factors, chromatin regulators, and kinases illustrate the utility of the dTAG system for drug target validation and discovery^6,12–23^. Despite this broad applicability, we observed context-specific and protein-specific differences in the effectiveness of dTAG-13 for inducing target protein degradation.

**Fig. 1:**
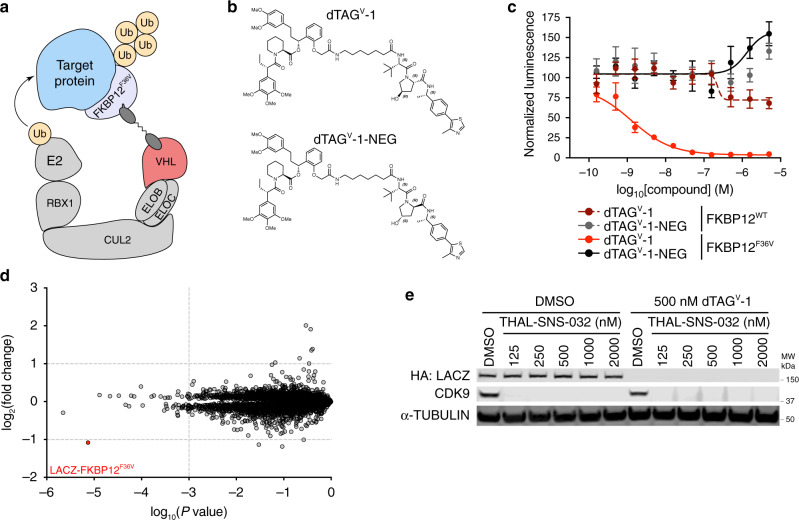
dTAG^V^-1 is an exclusively selective degrader of FKBP12^F36V^-tagged proteins. **a** Schematic depiction of the dTAG system using VHL-recruiting dTAG molecules. VHL-recruiting dTAG molecules promote ternary complex formation between the FKBP12^F36V^-tagged target protein and E3 ubiquitin ligase complex, inducing target protein ubiquitination and degradation. **b** Chemical structures of dTAG^V^-1 and dTAG^V^-1-NEG. **c** DMSO-normalized ratio of Nluc/Fluc signal of 293FT FKBP12^WT^-Nluc or FKBP12^F36V^-Nluc cells treated with dTAG^V^-1 or dTAG^V^-1-NEG for 24 h. Data are presented as mean ± s.d. of *n* = 4 biologically independent samples and are representative of *n* = 3 independent experiments. **d** Protein abundance after treatment of PATU-8902 LACZ-FKBP12^F36V^ clone with 500 nM dTAG^V^-1 for 4 h compared to DMSO treatment. Volcano plots depict fold change abundance relative to DMSO versus *P* value derived from a two-tailed Student’s *t*-test. Fold change values and significance designations derived from a two-tailed Student’s *t*-test and a permutation-based FDR estimation are provided in Supplementary Data 2. Data are from *n* = 3 biologically independent samples. **e** Immunoblot analysis of PATU-8902 LACZ-FKBP12^F36V^ clone co-treated with DMSO, THAL-SNS-032, and/or dTAG^V^-1 as indicated for 24 h. Data is representative of *n* = 3 independent experiments. Source data for **c** and **e** are provided as a Source Data file.

Here, we report the synthesis, characterization and utility of a second generation, in vivo-compatible dTAG molecule that recruits the von Hippel-Lindau (VHL) E3 ligase complex, dTAG^V^-1 (Fig. 1a, b). We demonstrate that dTAG^V^-1 degrades fusion proteins recalcitrant to CRBN-mediated degradation, exemplified by EWS/FLI, a driver of Ewing sarcoma. Collectively, this study describes an important extension to the dTAG platform, towards a universally applicable strategy for direct protein control.

## Results

### dTAG^V^-1 is a potent, selective, in vivo-compatible degrader

To identify a VHL-recruiting dTAG molecule, we synthesized ortho-AP1867-conjugated analogs with varying VHL-binding ligands and linker composition and screened for cellular activity in 293FT FKBP12^WT^-Nluc and FKBP12^F36V^-Nluc dual luciferase systems^6^ (Fig. 1b, c and Supplementary Fig. 1c, d), culminating in the identification of dTAG^V^-1 (Fig. 1b). Full structure activity relationships of our complete CRBN-recruiting and VHL-recruiting dTAG molecule series will be disclosed in a separate manuscript. dTAG^V^-1 induced potent degradation of FKBP12^F36V^-Nluc with no effects on FKBP12^WT^-Nluc, demonstrating that dTAG^V^-1 is an FKBP12^F36V^-selective degrader (Fig. 1c). dTAG^V^-1-NEG, a diastereomer of dTAG^V^-1 that can no longer bind and recruit VHL^24^ and functions as a heterobifunctional negative control, had no activity on FKBP12^F36V^-Nluc or FKBP12^WT^-Nluc (Fig. 1b, c). These effects were recapitulated in PATU-8902 LACZ-FKBP12^F36V^ cells^15^, corroborating effective degradation of FKBP12^F36V^-fusions in a pancreatic ductal adenocarcinoma (PDAC) context (Supplementary Fig. 1e). In both assays, levels of FKBP12^F36V^-fusion degradation with dTAG^V^-1 were comparable to dTAG-13 and dTAG-47^6,12,16^, two CRBN-recruiting dTAG molecules (Supplementary Fig. 1b–e). Use of dTAG^V^-1-NEG abrogated these effects comparably to the matched heterobifunctional negative control compounds that cannot bind and recruit CRBN^25,26^ (dTAG-13-NEG and dTAG-47-NEG) (Supplementary Fig. 1b–e). Multiplexed quantitative mass spectrometry-based proteomics demonstrated that LACZ-FKBP12^F36V^ was the only significantly degraded protein in the proteome (fold change < −2.0, *P* value < 0.001, FDR *q* < 0.05) upon treatment of PATU-8902 LACZ-FKBP12^F36V^ cells with 500 nM dTAG^V^-1, confirming the exquisite selectivity of the dTAG system (Fig. 1d and Supplementary Data 1, 2). No significant changes in protein levels of VHL or VHL-regulated targets were observed upon dTAG^V^-1 treatment, and no significantly degraded targets were observed with dTAG^V^-1-NEG (Supplementary Fig. 1f and Supplementary Data 1, 2).

**Fig. 2:**
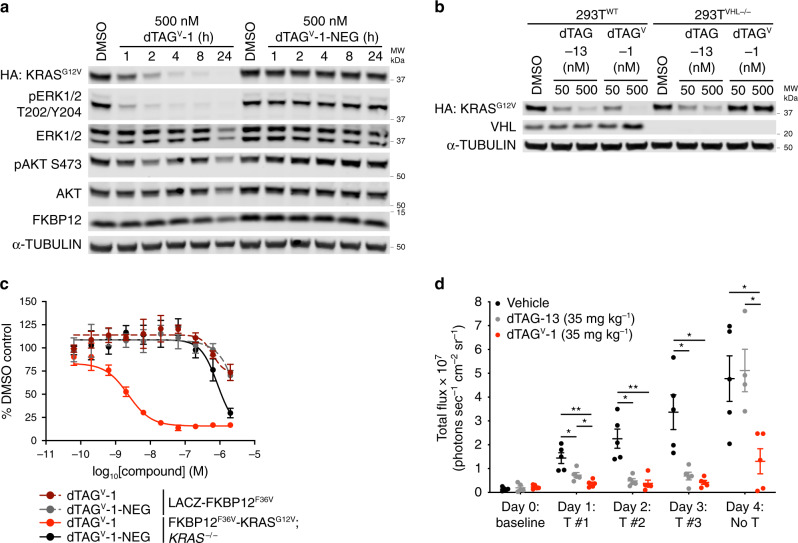
dTAG^V^-1 is an in vivo-compatible degrader of FKBP12^F36V^-tagged proteins. **a** Immunoblot analysis of PATU-8902 FKBP12^F36V^-KRAS^G12V^; *KRAS*^−/−^ clone treated with DMSO, dTAG^V^-1, or dTAG^V^-1-NEG for the indicated time-course. **b** Immunoblot analysis of 293T^WT^ FKBP12^F36V^-KRAS^G12V^ or 293T^VHL-/-^ FKBP12^F36V^-KRAS^G12V^ cells treated with DMSO or the indicated dTAG molecules for 24 h. Data in **a**, **b** are representative of *n* = 3 independent experiments. **c** DMSO-normalized antiproliferation of PATU-8902 LACZ-FKBP12^F36V^ or FKBP12^F36V^-KRAS^G12V^; *KRAS*^-/-^ clones treated with dTAG^V^-1 or dTAG^V^-1-NEG for 120 h. Cells were cultured as ultra-low adherent 3D-spheroid suspensions. Data are presented as mean ± s.d. of *n* = 4 biologically independent samples and are representative of *n* = 3 independent experiments. **d** Bioluminescent imaging to evaluate degradation of luciferase-FKBP12^F36V^ in mice was performed daily as follows: day 0 to establish baseline signal, day 1–3 to monitor luciferase-FKBP12^F36V^ signal 4 h after vehicle or dTAG molecule treatment (T), day 4 to monitor duration of luciferase-FKBP12^F36V^ signal 28 h after third and final vehicle or dTAG molecule treatment. Total flux for each mouse is depicted. Data are presented from vehicle (*n* = 5 biologically independent mice at day 0–4), dTAG-13 (*n* = 5 biologically independent mice at day 0–3; *n* = 4 biologically independent mice at day 4) or dTAG^V^-1 (*n* = 5 biologically independent mice at day 0–4) treated mice. *P* values are derived from a two-tailed Welch’s *t*-test (*P < 0.05, **P < 0.01) and are provided as a Source Data file. Source data for **a**–**d** are provided as a Source Data file.

We next evaluated the utility of dTAG^V^-1 for combinatorial degrader studies through co-treatment experiments with THAL-SNS-032, a previously reported CRBN-recruiting CDK9 degrader^27^. dTAG^V^-1 alone did not alter CDK9 protein levels (Fig. 1e, lane 7), which was confirmed by mass spectrometry-based proteomics analysis (Supplementary Data 2). dTAG^V^-1 effectively combined with THAL-SNS-032 and co-treatment led to pronounced degradation of both LACZ-FKBP12^F36V^ and CDK9 at levels comparable to those with treatment of each degrader alone (Fig. 1e, lanes 8–12), avoiding potential substrate competition effects^28^. To confirm the utility of dTAG^V^-1 for target-specific validation studies, we evaluated KRAS^G12V^ degradation, an oncogenic driver of PDAC, in PATU-8902 FKBP12^F36V^-KRAS^G12V^; *KRAS*^−/−^ cells^15^. dTAG^V^-1 treatment led to rapid KRAS^G12V^ degradation, which was not induced by dTAG^V^-1-NEG treatment (Fig. 2a and Supplementary Fig. 2a). KRAS^G12V^ degradation was rescued by pre-treatment with proteasome-inhibitor carfilzomib or Nedd8 activating enzyme inhibitor MLN4924, and by VHL knockout, consistent with the mechanism-of-action of a VHL-recruiting degrader (Fig. 2b and Supplementary Fig. 2b). We also observed the expected collapse in cellular signaling and diminished cell proliferation in 2D-monolayer and 3D-spheroid cultures upon FKBP12^F36V^-KRAS^G12V^ degradation with dTAG^V^-1 treatment, to levels comparable to CRBN-recruiting dTAG molecules (Fig. 2c and Supplementary Fig. 2c–e).

To confirm the in vivo applicability of dTAG^V^-1, we characterized the pharmacokinetic (PK) and pharmacodynamic (PD) profile of dTAG^V^-1 in mice. dTAG^V^-1 demonstrated improved properties compared to dTAG-13, with a longer half-life (*T*_1/2_ = 4.43, 2.41 h respectively) and greater exposure (AUC_inf_ = 18,517, 6140 h ng mL^−1^, respectively) by intraperitoneal administration at 10 mg kg^−1^ (Supplementary Table 1). To report on the PD profile of dTAG molecules, we employed MV4;11 luciferase-FKBP12^F36V^ (luc-FKBP12^F36V^) cells that allow noninvasive monitoring of bioluminescent signal upon dTAG molecule administration in mice^6^. Following tail vein injection of MV4;11 luc-FKBP12^F36V^ cells and establishment of leukemic burden, we performed daily bioluminescent measurements 4 h after vehicle, 35 mg kg^−1^ dTAG-13 or 35 mg kg^−1^ dTAG^V^-1 administration. Striking loss of bioluminescent signal was achieved 4 h after the first administration of dTAG^V^-1 (Fig. 2d and Supplementary Fig. 3). Consistent loss of bioluminescent signal was observed 4 h after each of the three dTAG-13 or dTAG^V^-1 administrations. Compared to dTAG-13, improved duration of degradation was also observed with dTAG^V^-1, with degradation evident 28 h after the final administration. These results support the use of dTAG^V^-1 as a potent and selective molecule to evaluate target-specific degradation in vitro and in vivo.

### dTAG^V^-1 enables EWS/FLI degradation in Ewing sarcoma

**Fig. 3:**
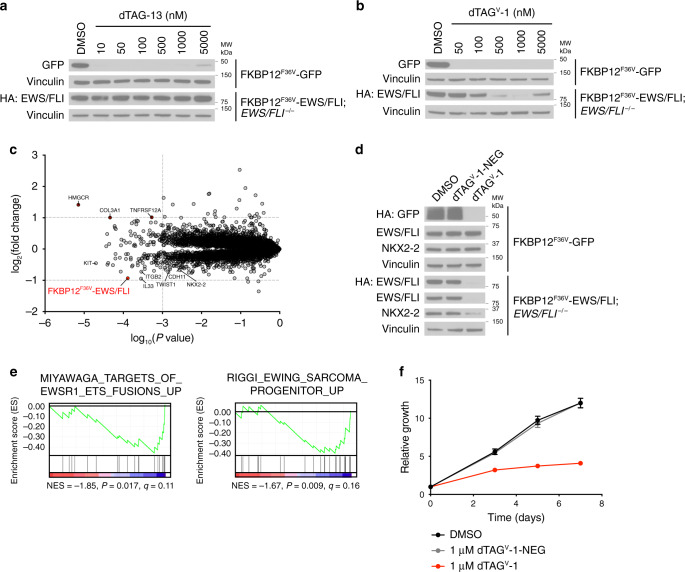
EWS/FLI degradation reverses abnormal proteomic signaling and proliferation. **a**, **b** Immunoblot analysis of EWS502 FKBP12^F36V^-GFP or FKBP12^F36V^-EWS/FLI; *EWS/FLI*^−/−^ cells treated with DMSO (**a**, **b**), dTAG-13 (**a**), or dTAG^V^-1 (**b**) for 24 h. Data in **a**, **b** are representative of *n* = 3 independent experiments. **c** Protein abundance after treatment of EWS502 FKBP12^F36V^-EWS/FLI; *EWS/FLI*^−/−^ cells with 1 μM dTAG^V^-1 for 6 h compared to DMSO treatment. Volcano plots depict fold change abundance relative to DMSO versus *P* value derived from a two-tailed Student’s *t*-test. Fold change values and significance designations derived from a two-tailed Student’s *t*-test and a permutation-based FDR estimation are provided in Supplementary Data 2. Data are from *n* = 2 for DMSO and *n* = 3 for dTAG^V^-1 biologically independent samples. **d** Immunoblot analysis of EWS502 FKBP12^F36V^-GFP or FKBP12^F36V^-EWS/FLI; *EWS/FLI*^−/−^ cells treated with DMSO, 1 μM dTAG^V^-1 or 1 μM dTAG^V^-1-NEG for 24 h. Data are representative of *n* = 3 independent experiments. **e** GSEA signatures upon assessment of significantly differentially expressed target proteins (FDR < 0.05) after treatment of EWS502 FKBP12^F36V^-EWS/FLI; *EWS/FLI*^−/−^ cells as described in **c**. Data are from *n* = 2 for DMSO and *n* = 3 for dTAG^V^-1 biologically independent samples. **f** Relative growth of EWS502 FKBP12^F36V^-EWS/FLI; *EWS/FLI*^−/−^ cells treated with DMSO, dTAG^V^-1, or dTAG^V^-1-NEG. *Y*-axis represent luminescence values relative to day 0. Data are presented as mean ± s.d. of *n* = 8 technical replicates and are representative of *n* = 3 independent experiments. Source data for **a**, **b**, **d**, and **f** are provided as a Source Data file.

There is long-standing interest in the oncology community in identifying targetable dependencies in Ewing sarcoma. Ewing sarcoma is driven by the translocation of *EWSR1* and members of the *ETS* transcription factors, most commonly *FLI*, giving rise to a fusion transcription factor oncoprotein that activates an aberrant transcriptional program^29^. Currently, there are a shortage of model systems and direct-acting agents that allow modulation of EWS/FLI levels or activity with precise kinetic control. To evaluate the effects of EWS/FLI degradation in Ewing sarcoma, we selected EWS502 cells, which are highly dependent on EWS/FLI for proliferation (Supplementary Fig. 4a). Through simultaneous expression of FKBP12^F36V^-EWS/FLI and Cas9/sgFLI, we developed FKBP12^F36V^-EWS/FLI; *EWS/FLI*^−/−^ cells (Supplementary Fig. 4b). FKBP12^F36V^-GFP was expressed in EWS502 cells as a control and was effectively degraded upon treatment with dTAG-13 and dTAG^V^-1, indicating the ability to co-opt both CRBN and VHL for targeted degradation in this cell line (Fig. 3a, b). The characteristic “hook effect” behavior^30^, which is commonly observed with heterobifunctional degraders^6^, was also evident at high doses (5000 nM) of dTAG molecule treatment due to saturation of FKBP12^F36V^ and E3 ubiquitin ligase binding sites. However, FKBP12^F36V^-EWS/FLI was only susceptible to degradation upon treatment with dTAG^V^-1 in EWS502 cells, which was evident within 1 h of treatment, highlighting the necessity of comparative evaluation of both CRBN-recruiting and VHL-recruiting dTAG molecules (Fig. 3a, b and Supplementary Fig. 4c). To confirm FKBP12^F36V^-EWS/FLI degradation and identify immediate downstream EWS/FLI targets, we performed acute quantitative mass spectrometry-based proteomics and observed pronounced loss of FKBP12^F36V^-EWS/FLI upon dTAG^V^-1 treatment (Fig. 3c and Supplementary Data 1, 2). dTAG^V^-1 led to a decrease in downstream EWS/FLI targets such as NKX2-2, and Gene Set Enrichment Analysis (GSEA) of the differentially regulated target proteins (FDR < 0.05) identified known EWS/FLI and Ewing sarcoma signatures (Fig. 3c–e). Potent antiproliferative effects were also observed upon EWS/FLI degradation at longer time-points, which were absent with dTAG^V^-1-NEG treatment and in FKBP12^F36V^-GFP cell lines (Fig. 3f and Supplementary Fig. 4d).

To evaluate the dependence of VHL-recruiting dTAG molecule activity on linker length and composition, we synthesized and evaluated dTAG-63, an analog of dTAG^V^-1. dTAG-63 is composed of a polyethylene glycol linker, (PEG)_3_, rather than an all carbon linker, between the FKBP12^F36V^ and E3 ubiquitin ligase binders (Supplementary Fig. 5a), and is a matched molecular pair to our previously disclosed first-generation CRBN-recruiting compound, dTAG-7^6,12^. Treatment of FKBP12^F36V^-EWS/FLI; *EWS/FLI*^−/−^ cells with dTAG-63 led to marginal degradation of FKBP12^F36V^-EWS/FLI, limited changes in NKX2-2 protein levels and modest antiproliferative effects relative to dTAG^V^-1 (Supplementary Fig. 5b, c). Similar trends in antiproliferation upon FKBP12^F36V^-KRAS^G12V^ degradation were observed upon treatment of PATU-8902 FKBP12^F36V^-KRAS^G12V^; *KRAS*^−/−^ cells with dTAG-63 (Supplementary Fig. 5d). These results are consistent with our prior observation that dTAG-7 is less effective across multiple cell lineages than dTAG-13, a CRBN-recruiting dTAG molecule with an all carbon linker^6^.

Finally, while we observed pronounced antiproliferative activity upon degradation of EWS/FLI with dTAG^V^-1, our data indicates that combination approaches may be necessary to achieve complete loss of viability. Prior work indicates that BET bromodomain inhibitors and degraders may have applications in Ewing Sarcoma^31^. To investigate potential synergy between direct and indirect repression of the EWS/FLI transcriptional program, we evaluated degradation of EWS/FLI in combination with BET bromodomain degradation. We observed that dBET6, a CRBN-recruiting, pan-BET bromodomain degrader^32^, synergized strongly with VHL-mediated EWS/FLI degradation (Supplementary Fig. 6a–c). Together, this data exemplifies the utility of VHL-recruiting dTAG molecules and provides model systems to evaluate the acute and prolonged consequences of EWS/FLI loss.

## Discussion

We report dTAG^V^-1, a potent and exclusively selective VHL-recruiting degrader of FKBP12^F36V^-tagged proteins. dTAG^V^-1 displays improved PK/PD properties and serves as an optimized tool for in vivo applications. Through evaluation of mutant KRAS degradation in PDAC models, we show that either CRBN or VHL can be co-opted to alleviate the aberrant signaling coordinated by this oncoprotein. By contrast, we observed contextual differences in the ability of these E3 ubiquitin ligase complexes to degrade EWS/FLI. This is consistent with our recent report demonstrating effective degradation of a core mediator subunit (MED14) with dTAG^V^-1 in HCT116 cells, a context in which CRBN-recruiting dTAG molecules were not effective^22^. We observed that rapid MED14 degradation abrogated lineage-specifying transcriptional circuits. Together, our studies provide support for use of dTAG^V^-1 to overcome the current limitations of the dTAG system, enabling evaluation of the direct effects of fusion proteins recalcitrant to CRBN-recruiting dTAG molecules.

Employing dTAG^V^-1 to study EWS/FLI, we demonstrate that VHL-mediated degradation of EWS/FLI rapidly alters downstream target protein expression and leads to pronounced growth defects in Ewing sarcoma cells, providing a powerful model system to investigate immediate consequences of EWS/FLI loss. This data supports that targeting EWS/FLI for degradation with direct-acting heterobifunctional degraders or molecular glues may be a tractable strategy and identifies potential combination strategies with BET bromodomain degraders. Together, the suite of dTAG molecules and paired controls provided in this study will facilitate evaluation of the functional consequences of precise posttranslational protein removal for an expanded target pool. The dTAG system enables rapid modulation of protein abundance and serves as a versatile strategy to determine whether targeted degradation is a promising drug development approach for a given target in vitro and in vivo.

## Methods

### Molecule synthesis

Full details on molecule synthesis are provided in Supplementary Methods and Supplementary Figs. 7–18.

### Cell lines

The following cell lines were employed in this study: 293T (source: ATCC #CRL-3216, media: DMEM with 10% FBS and 1% Penicillin–Streptomycin), 293FT (source: Thermo Fisher Scientific #R70007, media: DMEM with 10% FBS and 1% Penicillin–Streptomycin), PATU-8902 (source: DSMZ #ACC-179, media: DMEM with 10% FBS and 1% Penicillin–Streptomycin), MV4;11 (source: ATCC #CRL-9591, media: RPMI with 10% FBS and 1% Penicillin–Streptomycin) and EWS502 (source: kindly provided by Dr. Stephen L. Lessnick of Nationwide Children’s Hospital and established by Dr. Jonathan A. Fletcher of Harvard Medical School, media: RPMI with 15% FBS and 1% Penicillin–Streptomycin-l-Glutamine). Development of engineered cell lines are detailed below. All cell lines were maintained in 37 °C and 5% CO_2_ incubators and routinely tested negative for mycoplasma contamination using the MycoAlert Kit (Lonza).

### Lentiviral dTAG plasmid construction

To generate pLEX_305-dTAG-GFP and pLEX_305-dTAG-EWS/FLI plasmids, gateway recombination cloning strategies (Invitrogen) were employed to clone GFP or EWS/FLI into pLEX_305-N-dTAG^6^. In brief, to first generate pDONR221-EWS/FLI, EWS/FLI was cloned into pDONR221 using BP clonase (Invitrogen) after PCR with the following primers containing BP overhangs: Forward-N-E/F-dTAG, 5′-ggggacaagtttgtacaaaaaagcaggcttcgcgtccacggattacagtacct-3′ and Reverse-N-E/F-dTAG, 5′-ggggaccactttgtacaagaaagctgggtcctagtagtagctgcctaagtgtgaaggc-3′. Second, pENTREGFP2 (Addgene #22450) and pDONR221-EWS/FLI were cloned into pLEX_305-N-dTAG using LR clonase (Invitrogen). pLEX_305-dTAG-KRAS^G12V^ and pLEX_305-LACZ-dTAG plasmids were also employed in this study^6,15^.

### Lentiviral CRISPR/Cas9 plasmid construction

pXPR007-sgGFP and pXPR007-sgKRAS were employed in this study^15^. To generate lentiCRISPR v2-Blast-sgFLI_Ex9, sgFLI_Ex9 (5′-GCCTCACGGCGTGCAGGAAG-3′) was cloned into lentiCRISPR v2-Blast vector (Addgene #83480) using BsmbI restriction sites. The PAM motif of sgFLI_Ex9 is present in an intron, enabling cutting of the endogenous locus only with cDNA rescue.

### Development of engineered cell lines

To generate 293T^WT^ FKBP12^F36V^-KRAS^G12V^ and 293T^VHL−/−^ FKBP12^F36V^-KRAS^G12V^ cells, concentrated lentiviral supernatants^6^ were applied to 293T^WT^ and 293T^VHL−/−^ cells^33^ in the presence of 4 µg mL^−1^ polybrene and transduced cell lines were selected with 2 µg mL^−1^ puromycin. To generate EWS502 FKBP12^F36V^-GFP cells, EWS502 cells were transduced with pLEX_305-dTAG-GFP lentiviral supernatant and selected with 1 µg mL^−1^ puromycin. To generate EWS502 FKBP12^F36V^-EWS/FLI; *EWS/FLI*^−/−^ cells, EWS502 cells were co-transduced with pLEX_305-dTAG-EWS/FLI and lentiCRISPR v2-Blast-sgFLI_Ex9 lentiviral supernatants. Cells were then selected with both 1 µg mL^−1^ puromycin (pLEX_305-dTAG-EWS/FLI) and 5 µg mL^−1^ blasticidin (lentiCRISPR v2-Blast-sgFLI_Ex9). Knockout of endogenous EWS/FLI and the expression of exogenous FKBP12^F36V^-EWS/FLI were confirmed by immunoblot and pooled transduced cell populations were employed for this study. PATU-8902 LACZ-FKBP12^F36V^ and FKBP12^F36V^-KRAS^G12V^; *KRAS*^−/−^ clones were also employed in this study^15^.

### FKBP12^WT^ and FKBP12^F36V^ dual luciferase assay

Dual luciferase assays were performed using 293FT FKBP12^WT^-Nluc and FKBP12^F36V^-Nluc cells^6^. In brief, cells were plated at 2000 cells per well in 20 µL of appropriate media in 384-well white culture plates (Corning), allowed to adhere overnight, and 100 nL of compounds were added using a Janus Workstation pin tool (PerkinElmer) for 24 h at 37 °C. To evaluate Fluc signal, plates were brought to room temperature, 20 µL of Dual-Glo Reagent (Promega) was added for 10 min and luminescence was measured on an Envision 2104 plate reader (PerkinElmer). Subsequently, 20 µL of Dual-Glo Stop & Glo Reagent (Promega) was added for 10 min and luminescence was again measured to capture Nluc signal. DMSO-normalized ratios of Nluc/Fluc signal was analyzed and plotted using GraphPad PRISM v8^6^.

### Immunoblotting

PATU-8902 LACZ-FKBP12^F36V^ and FKBP12^F36V^-KRAS^G12V^; *KRAS*^−/−^ cells and 293T cells were lysed with RIPA buffer supplemented with cOmplete protease inhibitors (Roche), PhosSTOP phosphatase inhibitors (Roche), and 0.1% benzonase (Novagen). Proteins were separated using NuPAGE Bolt^TM^ Bis–Tris gels (Thermo Fisher Scientific) and transferred to nitrocellulose membranes, which were blocked with Odyssey Blocking Buffer (LI-COR) and incubated with primary antibodies overnight. Membranes were washed three times with TBS-T, incubated with the appropriate fluorescently labeled infrared secondary antibodies, washed again three times with TBS-T, and assessed using an Odyssey CLx Imager (LI-COR)^6,15^. EWS502 cells were lysed with Cell Lysis Buffer (Cell Signaling Technology) supplemented with cOmplete protease inhibitors (Roche). Proteins were separated using SDS-PAGE gels and transferred to PVDF membranes, which were blocked with 5% milk in TBS-T and incubated with primary antibodies overnight. Membranes were washed five times with TBS-T, incubated with the appropriate horseradish peroxidase-conjugated secondary antibodies, washed again five times with TBS-T, and imaged. The following primary antibodies were employed in this study: HA (Cell Signaling Technology #3724 and #2367, dilution at 1:1000), phospho-ERK1/2 T202/Y204 (Cell Signaling Technology #4370, dilution at 1:1000), ERK1/2 (Cell Signaling Technology #4696, dilution at 1:1000), phospho-AKT S473 (Cell Signaling Technology #4060, dilution at 1:1000), AKT (Cell Signaling Technology #2920, dilution at 1:1000), FKBP12 (Abcam #ab24373, dilution at 1:1000), GFP (Cell Signaling Technology #2555, dilution at 1:1000), FLI (Abcam #ab15289, dilution at 1:2000), NKX2-2 (Abcam #ab187375, dilution at 1:1000), GAPDH (Cell Signaling Technology #2118, dilution at 1:3000), Vinculin (Cell Signaling Technology #13901, dilution at 1:3000), β-Actin (Cell Signaling Technology #58169, dilution at 1:3000), and α-Tubulin (Cell Signaling Technology #3873, dilution at 1:5000). Species-specific fluorescently labeled infrared secondary antibodies including IRDye 680LT anti-Mouse IgG (LI-COR #926-68020, dilution at 1:5000), IRDye 800CW anti-Mouse IgG (LI-COR #926-32210, dilution at 1:5000), IRDye 680LT anti-Rabbit IgG (LI-COR #926-68021, dilution at 1:5000), and IRDye 800CW anti-Rabbit IgG (LI-COR #926-32211, dilution at 1:5000) and peroxidase-linked secondary antibodies including anti-Mouse IgG (Thermo Fisher Scientific #45000680, dilution at 1:5000) and anti-Rabbit IgG (Thermo Fisher Scientific #45000682, dilution at 1:5000) were employed as appropriate.

### Analysis of cell viability

Cell viability was assayed in 2D-adherent or ultra-low adherent 3D-spheroids using CellTiter-Glo (Promega)^6,15^. Luminescence was measured on an Envision 2104 plate reader (PerkinElmer) and Fluostar Omega Reader (BMG Labtech) and data was analyzed using GraphPad PRISM v8. Synergy assessments were performed using CellTiter-Glo (Promega) with the following modifications to the protocol described in the ref. ^34^. In brief, EWS502 cells were plated at 1000 cells per well in 50 µL of appropriate media in 384-well white culture plates (Corning) allowed to adhere overnight, and 100 nL of compounds were added using a Janus Workstation pin tool (PerkinElmer) for 72 h. Cell viability was measured by addition of 10 µL of CellTiter-glo (Promega), followed by incubation for 15 minutes at room temperature. Luminescence was measured on an Envision 2104 plate reader (PerkinElmer) and data was analyzed using GraphPad PRISM v8.

### Quantitative proteomics: materials

The following reagents were employed: Isobaric TMT reagents (Thermo Fisher Scientific), BCA protein concentration assay kit (Thermo Fisher Scientific), Empore-C18 material for in-house made StageTips (3 M), Sep-Pak cartridges (100 mg, Waters), solvents for Liquid chromatography (LC) (J.T. Baker), mass spectrometry (MS)-grade trypsin (Thermo Fisher Scientific), Lys-C protease (Wako), and cOmplete protease inhibitors (Millipore Sigma). Unless otherwise noted, all other chemicals were purchased from Thermo Fisher Scientific.

### Quantitative proteomics: MS sample processing

Cell pellets from PATU-8902 and EWS502 cells were lysed using 8 M urea, 200 mM 4-(2-hydroxyethyl)-1-piperazinepropanesulfonic acid (EPPS) at pH 8.5 with protease inhibitors. Samples were further homogenized and DNA was sheered via sonication using a probe sonicator (20 × 0.5 s pulses; level 3). Total protein was determined using a BCA assay and stored at −80 °C until future use. A total of 100 µg of protein was aliquoted for each condition and TMT channel for further downstream processing. Protein extracts were reduced using 10 mM dithiothreitol (DTT) for 30 min at room temperature. Next, samples were alkylated with 20 mM iodoacetamide for 45 min in the dark at room temperature. To facilitate the removal of incompatible reagents, proteins were precipitated using chloroform methanol. Briefly, to 100 µL of each sample, 400 µL of methanol was added, followed by 100 µL of chloroform with thorough vortexing. Next, 300 µL of HPLC grade water was added and samples were vortexed thoroughly. Each sample was centrifuged at 14,000 × *g* for 5 min at room temperature. The upper aqueous layer was removed and the protein pellet was washed twice with methanol and centrifuged at 14,000 × *g* for 5 min at room temperature. Protein pellets were resolubilized in 200 mM EPPS buffer and digested overnight with Lys-C (1:100, enzyme:protein ratio) at room temperature. The next day, trypsin (1:100 ratio) was added and incubated at 37 °C for an additional 6 h in a ThermoMixer set to 1000 RPM.

To each digested sample, 30% anhydrous acetonitrile was added and 100 µg of peptides were labeled using 200 µg of TMT reagent (TMT1-TMT11). Following labeling, a 5% hydroxylamine solution was used to quench excess TMT reagent. To equalize protein loading, a ratio check was performed by pooling 2 µg of each TMT-labeled sample. Samples were pooled and desalted using in-house packed C18 StageTips and analyzed by LC-MS/MS. Normalization factors were calculated from this label check, samples were mixed 1:1 across all TMT channels and desalted using a 100 mg Sep-Pak solid phase extraction cartridge. Eluted pooled peptides were further fractionated with basic-pH reverse-phase (bRP) HPLC using an Agilent 300 extend C18 column and collected into a 96 deep-well plate. Samples were consolidated into 24 fractions, and 12 nonadjacent fraction were desalted using StageTips prior to analyses using LC-MS/MS^35–37^.

### Quantitative proteomics: MS data acquisition

All mass spectrometry data was acquired using an Orbitrap Fusion mass spectrometer in-line with a Proxeon NanoLC-1000 UHPLC system. Peptides were separated using an in-house 100 µm capillary column packed with 40 cm of Accucore 150 resin (2.6 µm, 150 Å) (Thermo Fisher Scientific) using a 180 min LC gradient per fraction. Eluted peptides were acquired using synchronous precursor selection (SPS-MS3) method for TMT quantification^38^. Briefly, MS1 spectra were acquired at 120 K resolving power with a maximum of 50 ms in the Orbitrap. MS2 spectra were acquired by selecting the top ten most abundant features via collisional induced dissociation (CID) in the ion trap using an automatic gain control (AGC) of 15 K, quadrupole isolation width of 0.7 *m*/*z* and a maximum ion time of 100 ms. For MS3 acquisition, a synchronous precursor selection of ten fragment ions was acquired with an AGC of 150 K for 150 ms and a normalized collision energy of 55.

### Quantitative proteomics: MS data analysis

All acquired raw data were converted to mzXML using Raw File Reader (v3.0.77). Spectra were processed using Comet (2019.01.5)^39–41^, search results were filtered using the LDA function MASS Package in R, and data were processed using an in-house informatics pipeline^42–44^. Custom code was not developed in this study. Briefly, peptide spectral libraries were first filtered to a peptide false discovery rate (FDR) of less than 1% using linear discriminant analysis employing a target decoy strategy. Spectral searches were performed using a 2020 Uniprot Human database including canonical isoforms (96,788 total entries) fasta formatted database which included custom sequences for LACZ-FKBP12^F36V^ and FKBP12^F36V^-EWS/FLI, common contaminants, reversed sequences (Uniprot Human, 2020) and the following parameters: 50 ppm precursor tolerance, fully tryptic peptides, fragment ion tolerance of 0.9 Da and a static modification of TMT (+229.163 Da) on lysine and peptide N-termini, carbamidomethylation of cysteine residues (+57.021 Da) were set as static modifications, while oxidation of methionine residues (+15.995 Da) was set as a variable modification. Resulting peptides were further filtered to obtain a 1% protein FDR and proteins were collapsed into groups. Reporter ion intensities were adjusted to correct for impurities during synthesis of different TMT reagents according to the manufacturer’s specifications. For quantitation, a total sum signal-to-noise of all report ions of 100 was required for analysis, isolation specificity at the MS2 and MS3 level >0.50 and no missing values (11/11 TMT channels). Protein quantitative values were normalized (column normalization) so that the sum of the signal for all protein in each channel was equal to account for sample loading. Mass spectrometry-based proteomics raw data files have been deposited to the ProteomeXchange Consortium via the PRIDE^45,46^ partner repository (PXD018937). Gene Set Enrichment Analysis (GSEA)^47^ was performed as indicated in the figure legends.

### Animal studies: compound formulation

For IP injections, dTAG-13 and dTAG^V^-1 were formulated by dissolving into DMSO and then diluting with 20% solutol (Sigma): 0.9% sterile saline (Moltox) (w:v) with the final formulation containing 5% DMSO. Maximal solubility of 35 mg kg^−1^ and 40 mg kg^−1^ were observed for dTAG-13 and dTAG^V^-1, respectively. Formulations were stable at room temperature for 7 days. For IV injections, dTAG-13 and dTAG^V^-1 were formulated by dissolving into DMSO and then diluting with 5% solutol (Sigma): 0.9% sterile saline (Moltox) (w:v) with the final formulation containing 5% DMSO.

### Animal studies: pharmacokinetic (PK) evaluation

All procedures for PK studies were compliant with ethical regulations for animal testing and research, and were approved by and performed in accordance with standards of the Institute Animal Care and Use Committee (IACUC) at Scripps Florida. Housing conditions for mice at Scripps Florida were as follows: lights on = 7 AM, lights off = 7 PM, average ambient temperature = 72 °F, and average humidity = 45%. PK was assessed in 8-week-old C57BL/6J male mice (Jackson Laboratory, #000664) with blood collected at 0.08, 0.25, 0.5, 1, 2, 4, 6, and 8 h (2 mg kg^−1^ dTAG-13 intravenous (IV) tail vein, 10 mg kg^−1^ dTAG-13 intraperitoneal (IP), and 2 mg kg^−1^ dTAG^V^-1 IV tail vein administrations) and 0.25, 0.5, 1, 2, 4, 6, 8, 24 and 48 h (2 mg kg^−1^ dTAG^V^-1 IP and 10 mg kg^−1^ dTAG^V^-1 IP administrations). Plasma was generated by centrifugation and plasma concentrations were determined by LC-MS/MS following the mass transition 49600à340 AMU. PK parameters were calculated using Phoenix WinNonlin to determine peak plasma concentration (*C*_max_), oral bioavailability (%*F*), exposure (AUC), half-life (*t*_1/2_), clearance (CL), and volume of distribution (*V*_d_).

### Animal studies: pharmacodynamic (PD) evaluation

All procedures for PD studies were compliant with ethical regulations for animal testing and research, and were approved by and performed in accordance with standards of the IACUC at Dana–Farber Cancer Institute. Housing conditions for mice at Dana–Farber Cancer Institute were as follows: lights on = 6 AM, lights off = 6 PM, average ambient temperature = 72 °F, and average humidity = 35–55%. Evaluation of degradation of luciferase in vivo was performed using MV4;11 luc-FKBP12^F36V^ cells and bioluminescent measurements^6,48^. In brief, 250,000 viable MV4;11 luc-FKBP12^F36V^ cells were transplanted by tail-vein injection in 8-week-old immunocompromised female mice (NOD.Cg-*Prkdc*^*scid*^*Il2rg*^*tm1Wjl*^/SzJ, NSG; Jackson Laboratory, #005557). Bioluminescent measurements were performed following IP injection of 75 mg kg^−1^ d-Luciferin (Promega). Mice were then anesthetized with 2−3% isoflurane, imaged on an IVIS Spectrum (Caliper Life Sciences), and total body bioluminescence was determined using a standardized region of interest. Two and seven days after tail-vein injection, bioluminescent measurements were performed to confirm engraftment^48^. Detectable bioluminescent signal in the cohort was observed at day 7 and was used as the baseline signal for each mouse. For treatments, compounds were formulated as described above, administered via IP injection and bioluminescent measurements were performed daily as described in Fig. 2d, beginning 8 days after tail-vein injection.

### Statistical analysis

Information regarding center values, error bars, number of replicates or samples, number of independent experiments, and statistical analyses are described in the corresponding figure legends and data files. Experiments were not blinded nor randomized, and sample sizes were not predetermined using statistical analyses.

### Reporting summary

Further information on research design is available in the Nature Research Reporting Summary linked to this article.

## Supplementary information

Supplementary Information

Description of Additional Supplementary Files

Supplementary Data 1

Supplementary Data 2

Reporting Summary

## Data Availability

Mass spectrometry-based proteomics raw data files are provided in Supplementary Data 1 and have been deposited to the ProteomeXchange Consortium via the PRIDE partner repository with the dataset identifier PXD018937. Mass spectrometry-based proteomics processed data files underlying Figs. 1d and 3c and Supplementary Fig. 1f are provided in Supplementary Data 2. The source data underlying Figs. 1c, e, 2a–d, 3a, b, d, f and Supplementary Figs. 1c–e, 2a–e, 4a–d, 5b–d, 6a–c are provided as a Source Data file. Reagents are available upon request, following completion of a material transfer agreement, or at: http://graylab.dana-farber.org/probes.html. Source data are provided with this paper.

## Source data

Source Data

## References

[1] Lai AC, Crews CM (2017). Induced protein degradation: an emerging drug discovery paradigm. Nat. Rev. Drug Discov..

[2] Yesbolatova A, Tominari Y, Kanemaki MT (2019). Ligand-induced genetic degradation as a tool for target validation. Drug Discov. Today Technol..

[3] Bonger KM, Chen LC, Liu CW, Wandless TJ (2011). Small-molecule displacement of a cryptic degron causes conditional protein degradation. Nat. Chem. Biol..

[4] Buckley DL (2015). HaloPROTACS: use of small molecule PROTACs to induce degradation of HaloTag fusion proteins. ACS Chem. Biol..

[5] Chung HK (2015). Tunable and reversible drug control of protein production via a self-excising degron. Nat. Chem. Biol..

[6] Nabet B (2018). The dTAG system for immediate and target-specific protein degradation. Nat. Chem. Biol..

[7] Nishimura K, Fukagawa T, Takisawa H, Kakimoto T, Kanemaki M (2009). An auxin-based degron system for the rapid depletion of proteins in nonplant cells. Nat. Methods.

[8] Koduri V (2019). Peptidic degron for IMiD-induced degradation of heterologous proteins. Proc. Natl Acad. Sci. USA.

[9] Neklesa TK (2011). Small-molecule hydrophobic tagging-induced degradation of HaloTag fusion proteins. Nat. Chem. Biol..

[10] Fulcher, L. J. et al. An affinity-directed protein missile system for targeted proteolysis. *Open Biol.* **6**, 160255 (2016). 10.1098/rsob.160255 PMC5090066 27784791

[11] Clift D (2017). A method for the acute and rapid degradation of endogenous proteins. Cell.

[12] Erb MA (2017). Transcription control by the ENL YEATS domain in acute leukaemia. Nature.

[13] Boija A (2018). Transcription factors activate genes through the phase-separation capacity of their activation domains. Cell.

[14] Brunetti L (2018). Mutant NPM1 maintains the leukemic state through HOX expression. Cancer Cell.

[15] Ferguson FM (2020). Discovery of a selective inhibitor of doublecortin like kinase 1. Nat. Chem. Biol..

[16] Huang HT (2017). MELK is not necessary for the proliferation of basal-like breast cancer cells. Elife.

[17] Li M (2020). Human cytomegalovirus IE2 drives transcription initiation from a select subset of late infection viral promoters by host RNA polymerase II. PLoS Pathog..

[18] Sulahian R (2019). Synthetic lethal interaction of SHOC2 depletion with MEK inhibition in RAS-driven cancers. Cell Rep..

[19] Weintraub AS (2017). YY1 is a structural regulator of enhancer-promoter loops. Cell.

[20] Weissmiller AM (2019). Inhibition of MYC by the SMARCB1 tumor suppressor. Nat. Commun..

[21] Moser SC, Voerman JSA, Buckley DL, Winter GE, Schliehe C (2017). Acute pharmacologic degradation of a stable antigen enhances its direct presentation on MHC class I molecules. Front. Immunol..

[22] Jaeger, M. G. et al. Selective mediator-dependence of cell type-specifying transcription. *Nat. Genet.* Epub ahead of print. (2020). 10.1038/s41588-020-0635-0 PMC7610447 32483291

[23] Pinch, B. et al. Identification of a potent and selective covalent Pin1 inhibitor. *Nat. Chem. Biol.* Epub ahead of print. (2020). 10.1038/s41589-020-0550-9 PMC7442691 32483379

[24] Raina K (2016). PROTAC-induced BET protein degradation as a therapy for castration-resistant prostate cancer. Proc. Natl Acad. Sci. USA.

[25] Brand M (2019). Homolog-selective degradation as a strategy to probe the function of CDK6 in AML. Cell Chem. Biol..

[26] Huang HT (2018). A chemoproteomic approach to query the degradable kinome using a multi-kinase degrader. Cell Chem. Biol..

[27] Olson CM (2018). Pharmacological perturbation of CDK9 using selective CDK9 inhibition or degradation. Nat. Chem. Biol..

[28] Sperling AS (2019). Patterns of substrate affinity, competition, and degradation kinetics underlie biological activity of thalidomide analogs. Blood.

[29] Riggi N (2014). EWS-FLI1 utilizes divergent chromatin remodeling mechanisms to directly activate or repress enhancer elements in Ewing sarcoma. Cancer Cell.

[30] Douglass EF, Miller CJ, Sparer G, Shapiro H, Spiegel DA (2013). A comprehensive mathematical model for three-body binding equilibria. J. Am. Chem. Soc..

[31] Gollavilli PN (2018). EWS/ETS-driven ewing sarcoma requires BET bromodomain proteins. Cancer Res..

[32] Winter GE (2017). BET bromodomain proteins function as master transcription elongation factors independent of CDK9 recruitment. Mol. Cell.

[33] Gechijian LN (2018). Functional TRIM24 degrader via conjugation of ineffectual bromodomain and VHL ligands. Nat. Chem. Biol..

[34] Li Z (2020). Development and characterization of a Wee1 kinase degrader. Cell Chem. Biol..

[35] Navarrete-Perea J, Yu Q, Gygi SP, Paulo JA (2018). Streamlined tandem mass tag (SL-TMT) protocol: an efficient strategy for quantitative (Phospho)proteome profiling using tandem mass Tag-synchronous precursor selection-MS3. J. Proteome Res..

[36] Paulo JA (2014). Nicotine alters the proteome of two human pancreatic duct cell lines. JOP.

[37] Paulo, J. A. & Gygi, S. P. Nicotine-induced protein expression profiling reveals mutually altered proteins across four human cell lines. *Proteomics* **17**, 1600319 (2017). 10.1002/pmic.201600319 PMC5540149 27862958

[38] McAlister GC (2014). MultiNotch MS3 enables accurate, sensitive, and multiplexed detection of differential expression across cancer cell line proteomes. Anal. Chem..

[39] Eng JK, McCormack AL, Yates JR (1994). An approach to correlate tandem mass spectral data of peptides with amino acid sequences in a protein database. J. Am. Soc. Mass Spectrom..

[40] Eng JK (2015). A deeper look into Comet–implementation and features. J. Am. Soc. Mass Spectrom..

[41] Eng JK, Jahan TA, Hoopmann MR (2013). Comet: an open-source MS/MS sequence database search tool. Proteomics.

[42] Beausoleil SA, Villen J, Gerber SA, Rush J, Gygi SP (2006). A probability-based approach for high-throughput protein phosphorylation analysis and site localization. Nat. Biotechnol..

[43] Elias JE, Gygi SP (2007). Target-decoy search strategy for increased confidence in large-scale protein identifications by mass spectrometry. Nat. Methods.

[44] Huttlin EL (2010). A tissue-specific atlas of mouse protein phosphorylation and expression. Cell.

[45] Deutsch EW (2020). The ProteomeXchange consortium in 2020: enabling ‘big data’ approaches in proteomics. Nucleic Acids Res..

[46] Perez-Riverol Y (2019). The PRIDE database and related tools and resources in 2019: improving support for quantification data. Nucleic Acids Res..

[47] Subramanian A (2005). Gene set enrichment analysis: a knowledge-based approach for interpreting genome-wide expression profiles. Proc. Natl Acad. Sci. USA.

[48] Pikman Y (2016). Targeting MTHFD2 in acute myeloid leukemia. J. Exp. Med..

