# Supplementary material for: Rapid and direct control of target protein levels with VHL-recruiting dTAG molecules
Source: Nat Commun. 2020 Sep 18;11:4687. doi: 10.1038/s41467-020-18377-w (PMC7501296; doi:10.1038/s41467-020-18377-w)
Supplement: Supplementary file 1 — Supplementary Information [file 41467_2020_18377_MOESM1_ESM.pdf]

SUPPLEMENTARY INFORMATION

Rapid and direct control of target protein levels with VHL-recruiting dTAG molecules

Behnam Nabet[1,2,10]*, Fleur M. Ferguson[1,2,10], Bo Kyung A. Seong[3,4], Miljan Kuljanin[5], Alan L. Leggett[1], Mikaela L. Mohardt[1], Amanda Robichaud[3], Amy S. Conway[3], Dennis L. Buckley[6,7], Joseph D. Mancias[5], James E. Bradner[6,7,8], Kimberly Stegmaier[3,4,9], Nathanael S. Gray[1,2]*

[1]Department of Cancer Biology, Dana-Farber Cancer Institute, Boston, Massachusetts, USA.
[2]Department of Biological Chemistry and Molecular Pharmacology, Harvard Medical School, Boston, Massachusetts, USA.
[3]Department of Pediatric Oncology, Dana-Farber Cancer Institute, Boston, Massachusetts, USA.
[4]The Broad Institute of MIT and Harvard, Cambridge, Massachusetts, USA.
[5]Division of Radiation and Genome Stability, Department of Radiation Oncology, Dana-Farber Cancer Institute, Boston, Massachusetts, USA.
[6]Department of Medical Oncology, Dana-Farber Cancer Institute, Boston, Massachusetts, USA.
[7]Present address: Novartis Institutes for BioMedical Research, Cambridge, Massachusetts, USA.
[8]Department of Medicine, Harvard Medical School, Boston, Massachusetts, USA.
[9]Division of Pediatric Hematology/Oncology, Boston Children's Hospital, Boston, Massachusetts, USA.
[10]These authors contributed equally to this work.
*Corresponding author

Correspondence may be addressed to:

Behnam Nabet
behnam_nabet@dfci.harvard.edu

Nathanael S. Gray
nathanael_gray@dfci.harvard.edu

SUPPLEMENTARY TABLES

		dTAG-13		$dTAG^V$-1		
**Parameter**	**Unit**	**IV**	**IP**	**IV**	**IP**	**IP**
Dose	mg kg^{-1}	2	10	2	2	10
T_{max}	hr	0.08	2.00	0.08	1.67	2.00
$T_{1/2}$	hr	1.46	2.41	3.02	3.64	4.43
C_{max}	ng mL^{-1}	2373	1263	7780	595	2123
AUC_{last}	hr*ng mL^{-1}	1242	5619	3245	2245	18088
AUC_{inf}	hr*ng mL^{-1}	1253	6140	3329	3136	18517
CL	ml min^{-1} kg^{-1}	32.5	28	10.1	10.7	9.05
V_{ss}	L kg^{-1}	1.8	-	0.56	-	-
F^b	%	-	-	-	-	-

Supplementary Table 1 | Table summarizing pharmacokinetic assessment of dTAG-13 and $dTAG^V$-1 by intraperitoneal (IP) or intravenous (IV) administration. Data are from *n* = 3 biologically independent mice.

SUPPLEMENTARY FIGURES

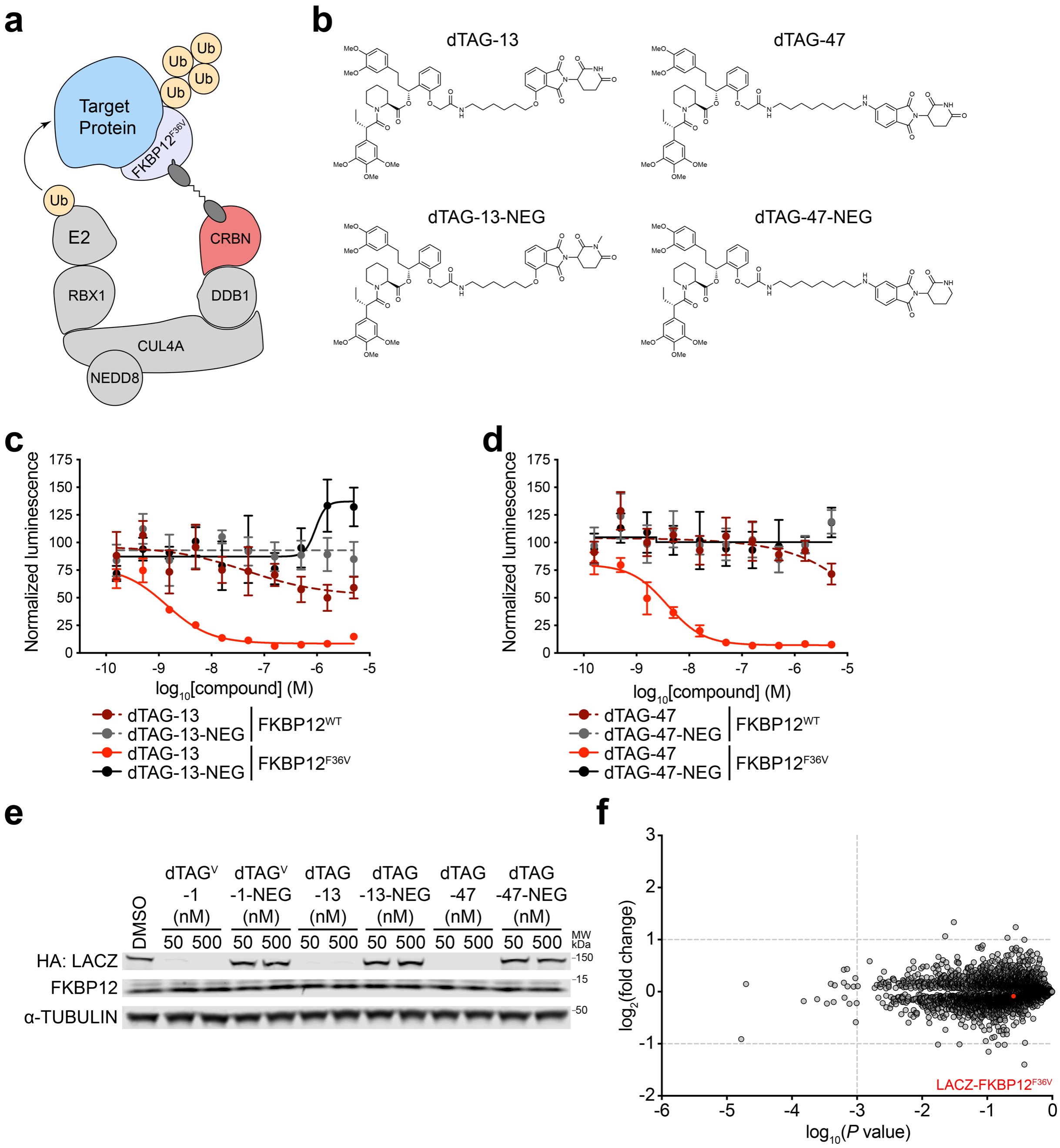

Supplementary Figure 1 | Evaluation of CRBN-recruiting dTAG molecules and heterobifunctional control compounds. (**a**) Schematic depiction of the dTAG system using CRBN-recruiting dTAG molecules. CRBN-recruiting dTAG molecules promote ternary complex formation between the $FKBP12^{F36V}$-tagged target protein and E3 ubiquitin ligase complex, inducing target protein ubiquitination and degradation. (**b**) Chemical structures of dTAG-13, dTAG-13-NEG, dTAG-47, and dTAG-47-NEG. (**c-d**) DMSO-normalized ratio of Nluc/Fluc signal of 293FT $FKBP12^{WT}$-Nluc or $FKBP12^{F36V}$-Nluc cells treated with the indicated dTAG molecules for 24 h. Data in **c-d** presented as mean ± s.d. of n = 4 biologically independent samples and

are representative of n = 3 independent experiments. (**e**) Immunoblot analysis of PATU-8902 LACZ-$FKBP12^{F36V}$ clone treated with DMSO or the indicated dTAG molecules for 4 h. Data are representative of n = 3 independent experiments. (**f**) Protein abundance after treatment of PATU-8902 LACZ-$FKBP12^{F36V}$ clone with 500 nM $dTAG^{V}$-1-NEG for 4 h compared to DMSO treatment. Volcano plots depict fold change abundance relative to DMSO versus P value derived from a two-tailed Student's t-test. Fold change values and significance designations derived from a two-tailed Student's t-test and a permutation-based FDR estimation are provided in Supplementary Data 2. Data are from n = 3 biologically independent samples. Source data for **c**-**e** are provided as a Source Data file.

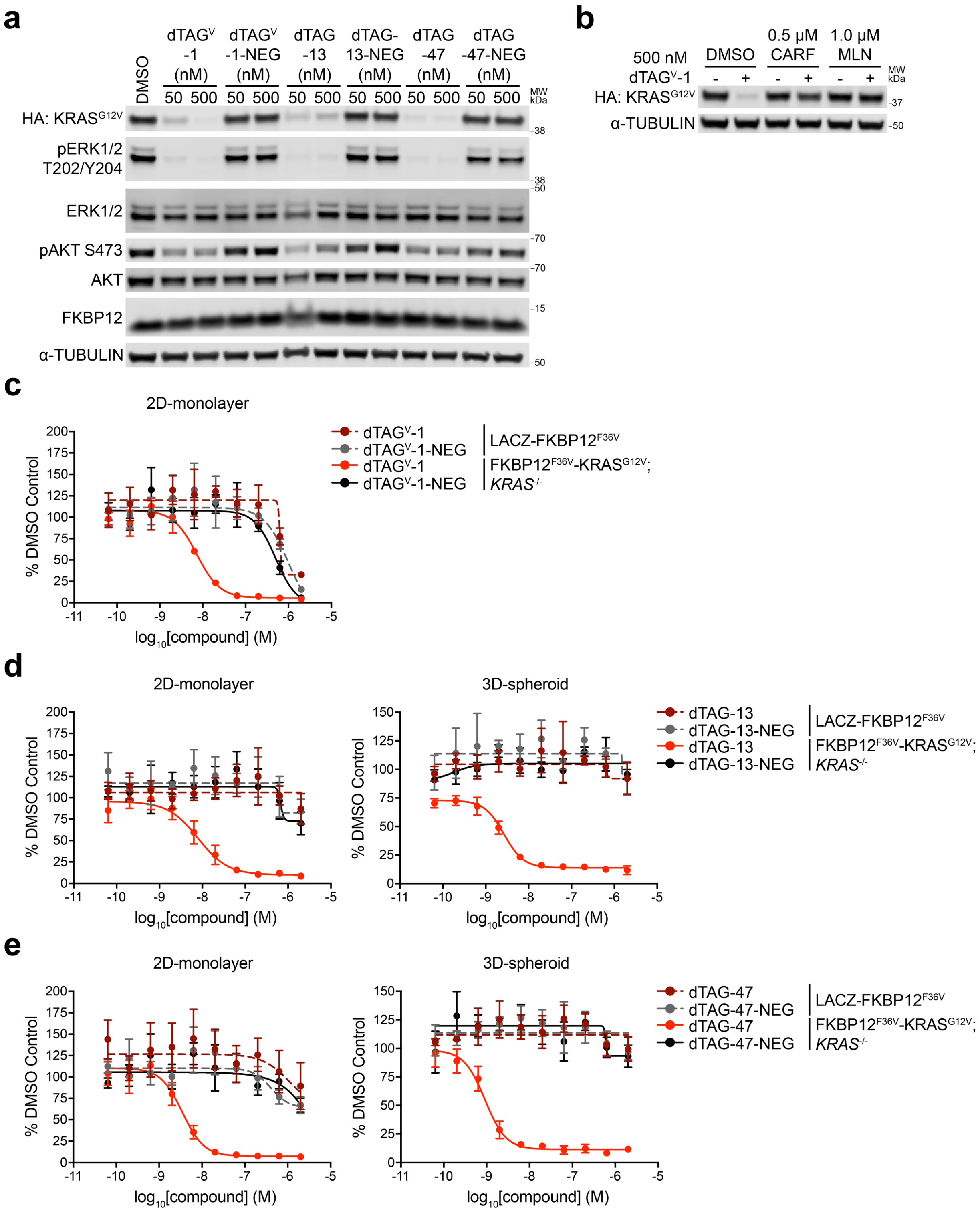

Supplementary Figure 2 | Mutant KRAS degradation diminishes aberrant signaling and viability. (**a**) Immunoblot analysis of PATU-8902 FKBP12^{F36V}-KRAS^{G12V}; *KRAS*$^{-/-}$ clone treated with DMSO or the indicated dTAG molecules for 4 h. (**b**) Immunoblot analysis of PATU-8902 FKBP12^{F36V}-KRAS^{G12V}; *KRAS*$^{-/-}$ clone pretreated with DMSO, Carfilzomib (CARF) or MLN4924

(MLN) for 2 h prior to DMSO or dTAG^{V}-1 treatment for 4 h. Data in **a**-**b** are representative of *n* = 3 independent experiments. (**c**-**e**) DMSO-normalized antiproliferation of PATU-8902 LACZ-FKBP12^{F36V} or FKBP12^{F36V}-KRAS^{G12V}; *KRAS*$^{-/-}$ clones treated with the indicated dTAG molecules for 120 h. Cells were cultured as 2D-monolayers or as ultra-low adherent 3D-spheroid suspensions as indicated. Data in **c**-**e** presented as mean ± s.d. of *n* = 4 biologically independent samples and are representative of *n* = 3 independent experiments. Source data for **a**-**e** are provided as a Source Data file.

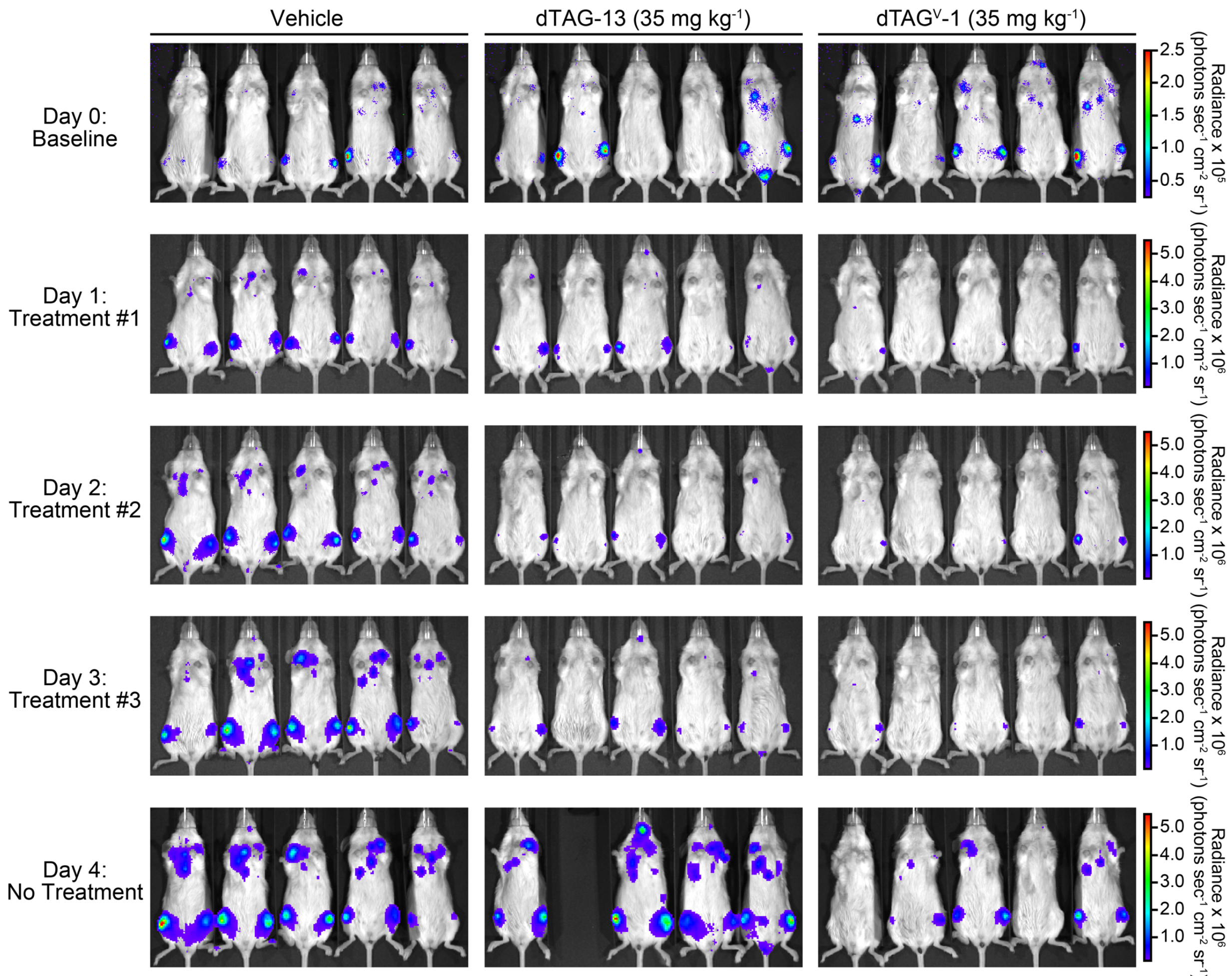

Supplementary Figure 3 | dTAG^{V}-1 induces degradation in vivo. Bioluminescence images of vehicle (*n* = 5 biologically independent mice at day 0-4), dTAG-13 (*n* = 5 biologically independent mice at day 0-3; *n* = 4 biologically independent mice at day 4) or dTAG^{V}-1 (*n* = 5 biologically independent mice at day 0-4) treated mice. The same mouse is shown on the same scale at each time point. Quantifications of total flux are provided in Fig. 2d.

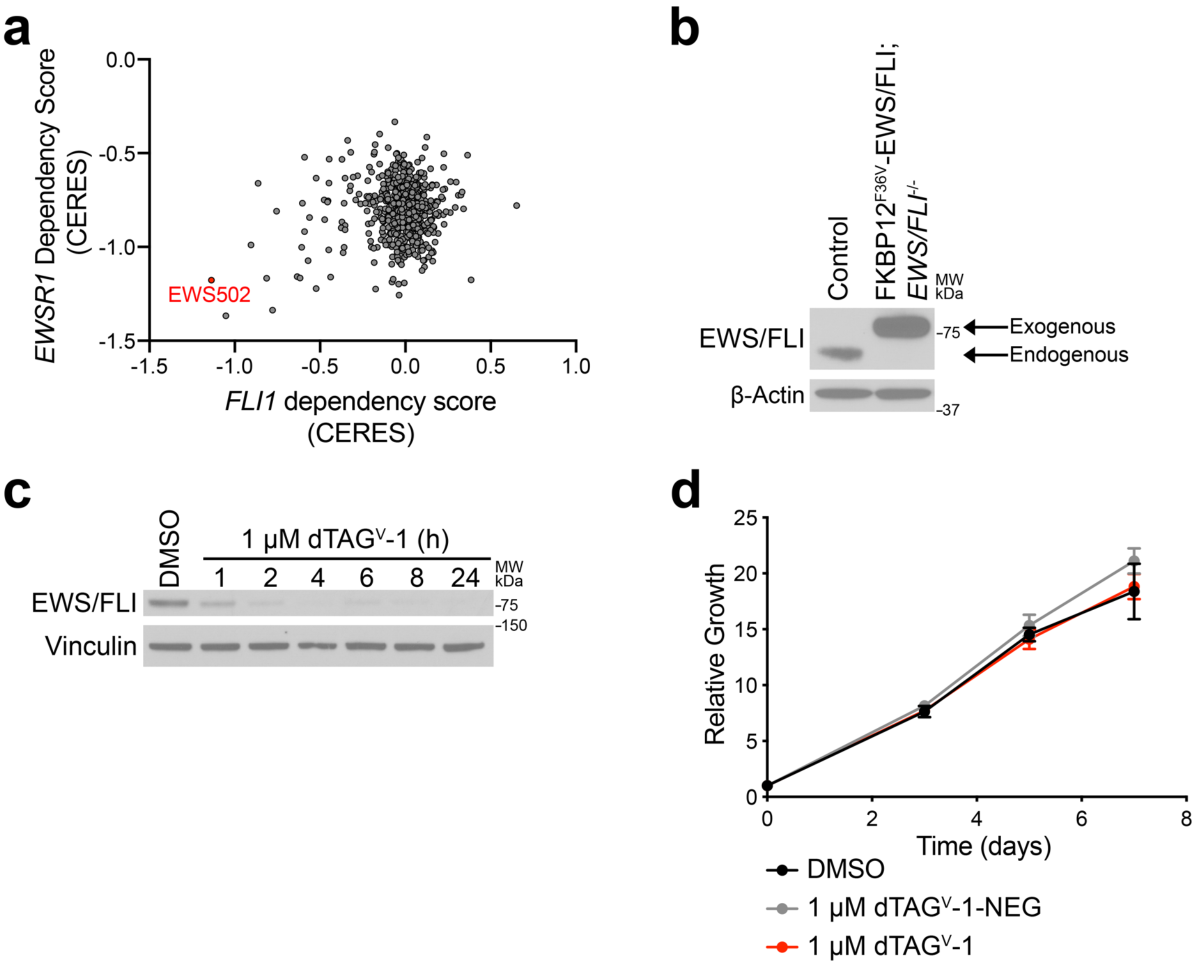

Supplementary Figure 4 | Evaluation of degradation with dTAG^{V}-1 in Ewing sarcoma cell lines. (**a**) Dependency score of *EWSR1* and *FLI1* from CRISPR (Avana) datasets from the Cancer Dependency Map portal. (**b**) Immunoblot analysis of EWS502 control and FKBP12^{F36V}-EWS/FLI; *EWS/FLI*$^{-/-}$ cells. (**c**) Immunoblot analysis of EWS502 FKBP12^{F36V}-EWS/FLI; *EWS/FLI*$^{-/-}$ cells treated with DMSO or dTAG^{V}-1 for the indicated time-points. Data in **b-c** are representative of *n* = 2 independent experiments. (**d**) Relative growth of EWS502 FKBP12^{F36V}-GFP cells treated with DMSO or the indicated dTAG molecules. Y-axis represent luminescence values relative to day 0. Data are presented as mean ± s.d. of *n* = 8 technical replicates and are representative of *n* = 3 independent experiments. Source data for **a-d** are provided as a Source Data file.

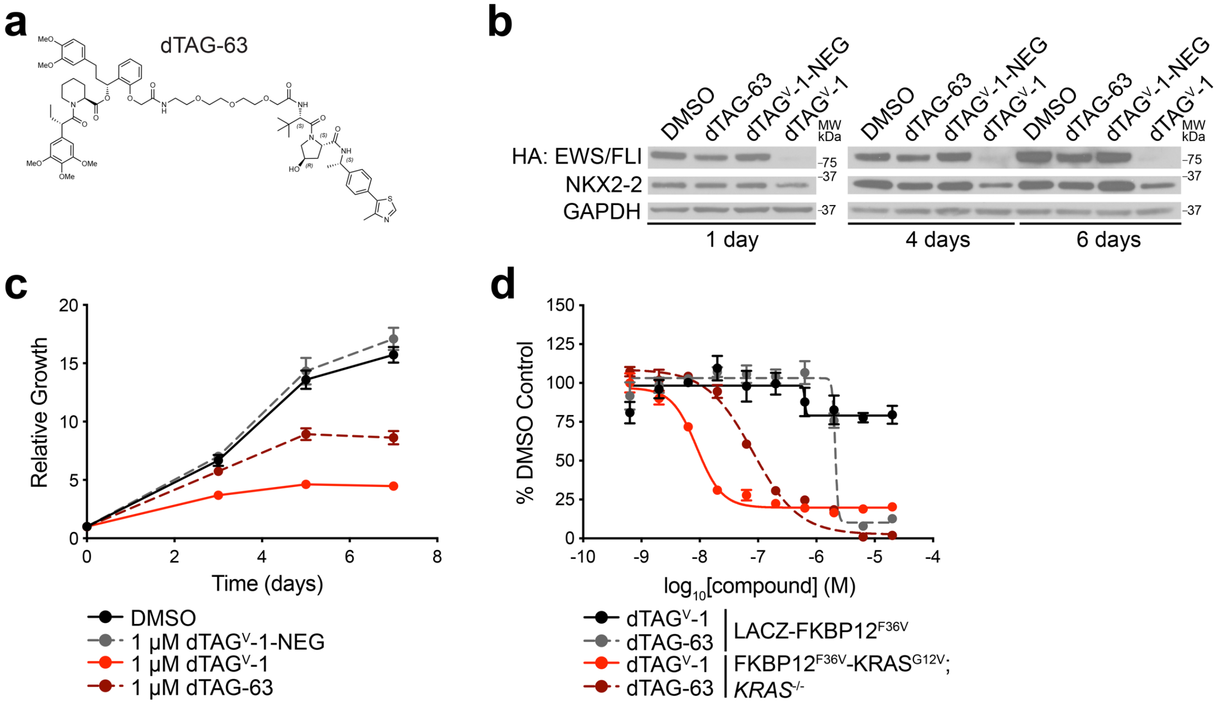

Supplementary Figure 5 | dTAG^{V}-1 displays improved potency and duration of degradation than dTAG-63. (**a**) Chemical structure of dTAG-63. (**b**) Immunoblot analysis of EWS502 FKBP12^{F36V}-EWS/FLI; *EWS/FLI*$^{-/-}$ cells treated with DMSO or the indicated dTAG molecules for the indicated time-points. Data are representative of n = 2 independent experiments. (**c**) Relative growth of EWS502 FKBP12^{F36V}-EWS/FLI; *EWS/FLI*$^{-/-}$ cells treated with DMSO or the indicated dTAG molecules. Y-axis represent luminescence values relative to day 0. Data are presented as mean ± s.d. of n = 8 technical replicates and are representative of n = 3 independent experiments. (**d**) DMSO-normalized antiproliferation of PATU-8902 LACZ-FKBP12^{F36V} or FKBP12^{F36V}-KRAS^{G12V}; *KRAS*$^{-/-}$ clones treated with the indicated dTAG molecules for 120 h. Cells were cultured as 2D-monolayers. Data are presented as mean ± s.d. of n = 4 biologically independent samples and are representative of n = 3 independent experiments. Source data for **b-d** are provided as a Source Data file.

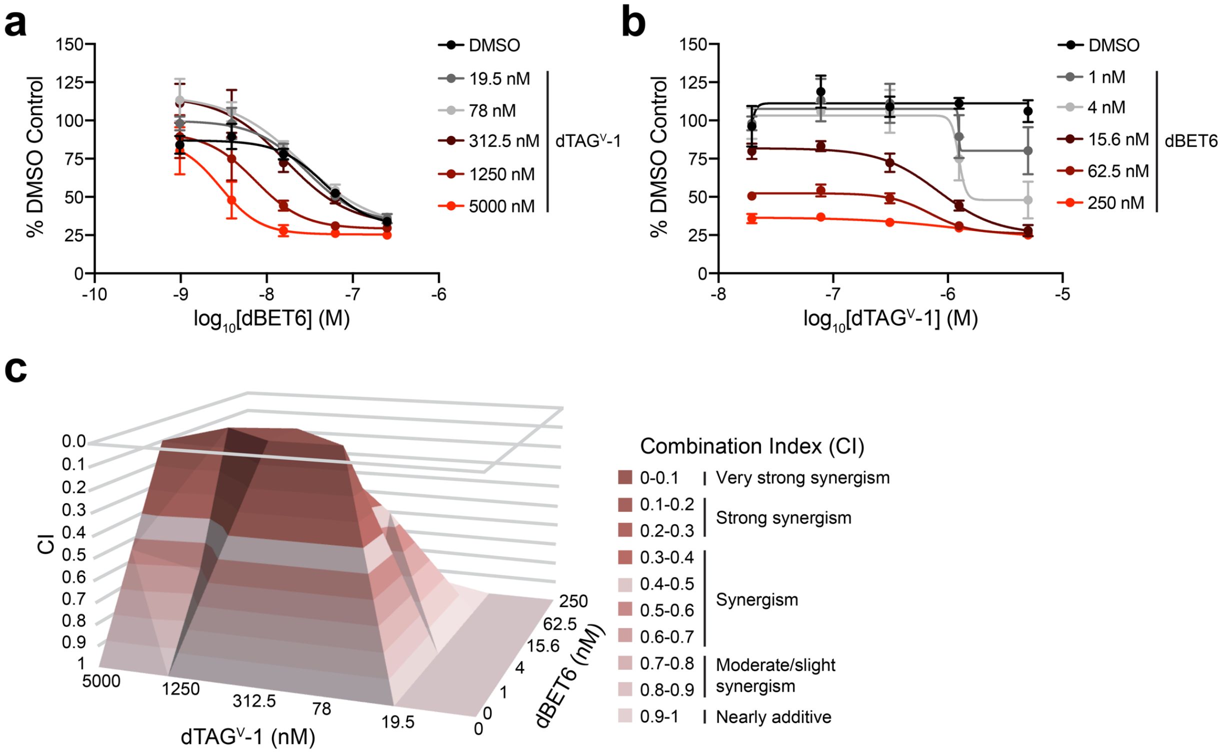

Supplementary Figure 6 | EWS/FLI degradation synergizes with BET bromodomain degradation. (**a-c**) Antiproliferation of EWS502 FKBP12^{F36V}-EWS/FLI; *EWS/FLI*$^{-/-}$ cells co-treated with the indicated combinations of dTAG^{V}-1 or dBET6 for 72 h. Plots in **a-b** depict DMSO-normalized antiproliferation. Plot in **c** depicts combination index (CI) scores. Data in **a-c** are presented from *n* = 4 biologically independent samples and are representative of *n* = 3 independent experiments. Source data for **a-c** are provided as a Source Data file.

SUPPLEMENTARY METHODS

Molecule synthesis

General methods

Unless otherwise noted, reagents and solvents were obtained from commercial suppliers and were used without further purification. ^1H NMR spectra were recorded on 500 MHz Bruker Avance III spectrometer, and chemical shifts are reported in parts per million (ppm, δ) downfield from tetramethylsilane (TMS). Coupling constants (J) are reported in Hz. Spin multiplicities are described as s (singlet), br (broad singlet), d (doublet), t (triplet), q (quartet), and m (multiplet). Mass spectra were obtained on a Waters Acquity UPLC. Preparative HPLC was performed on a Waters Sunfire C18 column (19 mm × 50 mm, 5 μM) using a gradient of 15−95% methanol in water containing 0.05% trifluoroacetic acid (TFA) over 22 min (28 min run time) at a flow rate of 20 mL/min. Assayed compounds were isolated and tested as TFA salts. Purities of assayed compounds were in all cases greater than 95%, as determined by reverse-phase HPLC analysis. High resolution masses were analyzed on a Thermo q-Exactive Plus mass spectrometer coupled to an Ultimate 3000 uHPLC. The samples were analyzed using flow injection analysis directly into the mass spectrometer with 50% acetonitrile/0.1% formic acid in water. The instrument was calibrated for m/z scale immediately prior to analysis. All analytes were detected and formula was confirmed with better than 2 ppm mass accuracy.

Compounds Ortho-AP acid,[2] dTAG-13,[3] dTAG-47,[4] THAL-SNS-032,[5] and dBET6[6] are commercially available. For the studies described here we utilized compounds stocks previously synthesized in-house, following the protocols which can be found in published literature and reaction schemes found on the Gray lab website. Synthesis of all newly created molecules used in this study is described below.

Synthesis of dTAG^{V}-1 and dTAG^{V}-1-NEG

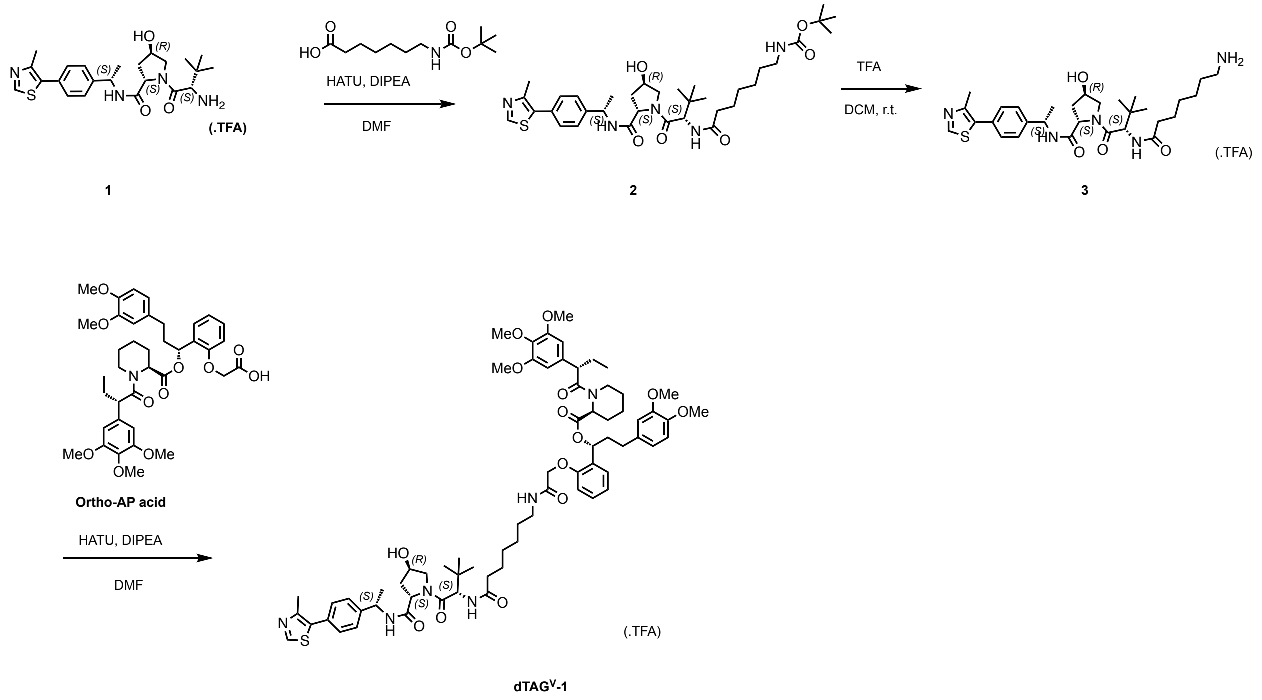

Supplementary Figure 7 | Scheme used for synthesis of dTAG^{V}-1 (Scheme 1)

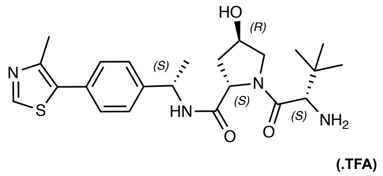

Supplementary Figure 8 | (2*S*,4*R*)-1-((*S*)-2-amino-3,3-dimethylbutanoyl)-4-hydroxy-*N*-((*S*)-1-(4-(4-methylthiazol-5-yl)phenyl)ethyl)pyrrolidine-2-carboxamide (1)

The title compound was prepared with minor modifications to the protocol previously described[1]. Specifically trifluoroacetic acid (4N in DCM) was used in place of hydrochloric acid (4N in MeOH) to afford the title compound in quantitative yield.

^{1}H NMR (500 MHz, DMSO-d_6) δ 9.00 (s, 1H), 8.60 (dd, J = 38.0, 7.8 Hz, 1H), 8.01 (dd, J = 16.6, 5.4 Hz, 3H), 7.45 (dd, J = 8.4, 2.9 Hz, 2H), 7.39 (dd, J = 8.3, 4.9 Hz, 2H), 5.62 (s, 1H), 4.94 (td, J = 7.2, 2.6 Hz, 1H), 4.55 (td, J = 9.5, 7.5 Hz, 1H), 4.35 (s, 1H), 3.93 (d, J = 5.5 Hz, 1H), 3.68 (d, J = 11.1 Hz, 1H), 3.51 (dd, J = 11.0, 3.8 Hz, 1H), 2.46 (s, 3H), 2.17 – 2.07 (m, 1H), 1.84 – 1.75 (m, 1H), 1.42 – 1.36 (m, 3H), 1.04 (d, J = 2.9 Hz, 9H).

LC/MS (ESI$^+$): *m/z* 445[M+H$^+$]

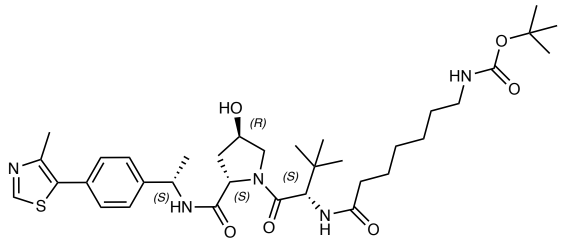

Supplementary Figure 9 | *tert*-butyl (7-(((*S*)-1-((2*S*,4*R*)-4-hydroxy-2-(((*S*)-1-(4-(4-methylthiazol-5-yl)phenyl)ethyl)carbamoyl)pyrrolidin-1-yl)-3,3-dimethyl-1-oxobutan-2-yl)amino)-7-oxoheptyl)carbamate (2)

The title compound was prepared with minor modifications to described protocols[1]. Briefly, to a stirred solution of 7-((*tert*-butoxycarbonyl)amino)heptanoic acid (36 mg, 0.12 mmol), HATU (46 mg, 0.12 mmol) and DIPEA (35 µL) in DMF was added **1** (50 mg, 0.1 mmol). The reaction mixture was stirred at room temperature for 16 h. The reaction mixture was diluted with sat. aq. sodium bicarbonate (20 mL) and extracted with EtOAc (3 x 50 mL). The residue was purified by flash chromatography to afford the title compound. (45 mg, 67 %).

^{1}H NMR (500 MHz, Methanol-d_4) δ 8.89 (s, 1H), 8.56 (dd, J = 8.0, 4.7 Hz, 1H), 8.00 (s, 1H), 7.48 – 7.42 (m, 4H), 6.56 (s, 1H), 5.51 (s, 1H), 5.07 – 4.98 (m, 1H), 4.64 (d, J = 8.9 Hz, 1H), 4.59 (dd, J = 9.0, 7.7 Hz, 1H), 4.45 (dp, J = 4.4, 2.0 Hz, 1H), 3.90 (dt, J = 11.2, 1.8 Hz, 1H), 3.80 – 3.74 (m, 2H), 3.05 (d, J = 7.0 Hz, 2H), 2.83 (s, 3H), 2.32 – 2.25 (m, 2H), 2.25 – 2.17 (m, 2H), 1.53 (d, J = 7.0 Hz, 3H), 1.45 (s, 9H), 1.41 – 1.30 (m, 8H), 1.06 (s, 9H).

LC/MS (ESI$^+$): *m/z* 672[M+H$^+$]

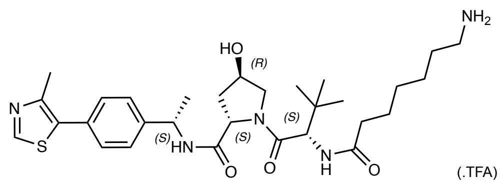

Supplementary Figure 10 | (2*S*,4*R*)-1-((*S*)-2-(7-aminoheptanamido)-3,3-dimethylbutanoyl)-4-hydroxy-*N*-((*S*)-1-(4-(4-methylthiazol-5-yl)phenyl)ethyl)pyrrolidine-2-carboxamide (3)

The title compound was prepared with minor modifications to described protocols[1]. Briefly, compound **2** (45 mg, 0.067 mmol) was dissolved in 4N TFA in DCM and stirred at room temperature for 2 h. The reaction mixture was concentrated *in vacuo* to afford the title compound (45 mg, quant), which was used without further purification.

LC/MS (ESI$^+$): *m/z* 572[M+H$^+$]

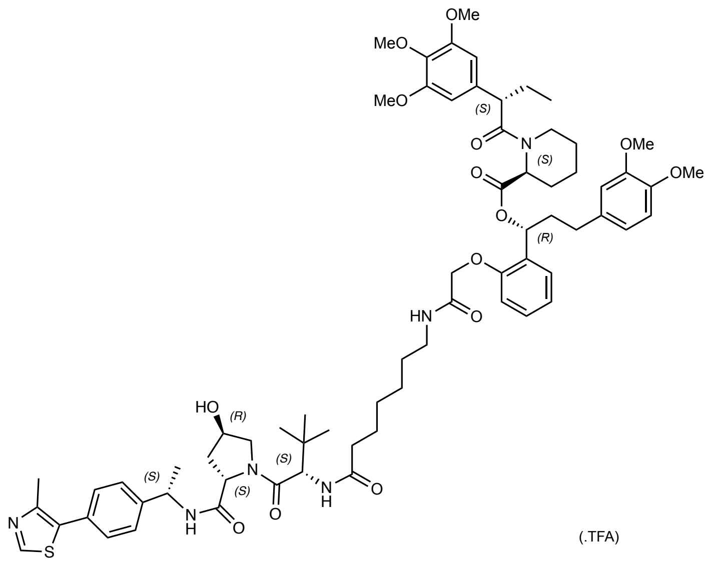

Supplementary Figure 11 | (*R*)-3-(3,4-dimethoxyphenyl)-1-(2-(2-((7-(((*S*)-1-((2*S*,4*R*)-4-hydroxy-2-(((*S*)-1-(4-(4-methylthiazol-5-yl)phenyl)ethyl)carbamoyl)pyrrolidin-1-yl)-3,3-dimethyl-1-oxobutan-2-yl)amino)-7-oxoheptyl)amino)-2-oxoethoxy)phenyl)propyl (*S*)-1-((*S*)-2-(3,4,5-trimethoxyphenyl)butanoyl)piperidine-2-carboxylate (dTAG^{V}-1)

To a stirred solution of ortho-AP acid (56 mg, 0.08 mmol), HATU (31 mg, 0.08 mmol), DIPEA (55 μL, 0.2 mmol) in DMF (2 mL) was added **3** (40 mg, 0.067 mmol). The reaction mixture was stirred for 16 h at room temperature, filtered and purified by HPLC to afford the title compound (34 mg, 41 %).

^{1}H NMR (500 MHz, DMSO-d_6) δ 8.99 (s, 1H), 8.37 (d, *J* = 7.8 Hz, 1H), 7.77 (dd, *J* = 9.3, 3.3 Hz, 1H), 7.70 (t, *J* = 5.8 Hz, 1H), 7.47 – 7.42 (m, 2H), 7.38 (d, *J* = 8.3 Hz, 2H), 7.21 (ddd, *J* = 8.5, 6.7, 2.4 Hz, 1H), 6.87 (d, *J* = 8.3 Hz, 1H), 6.83 – 6.79 (m, 2H), 6.75 (d, *J* = 2.0 Hz, 1H), 6.64 (dd, *J* = 8.2, 2.0 Hz, 1H), 6.62 (s, 1H), 6.56 (s, 2H), 6.03 (dd, *J* = 8.3, 4.9 Hz, 1H), 5.76 (s, 1H), 5.36 – 5.31 (m, 1H), 5.10 (d, *J* = 3.5 Hz, 1H), 4.92 (p, *J* = 7.3 Hz, 1H), 4.62 – 4.39 (m, 4H), 4.29 (d, *J* = 4.4 Hz, 1H), 4.06 (d, *J* = 13.6 Hz, 1H), 3.91 – 3.84 (m, 1H), 3.75 (s, 1H), 3.72 (s, 3H), 3.71 (s, 2H), 3.70 (s, 3H), 3.64 (d, *J* = 2.6 Hz, 1H), 3.61 (d, *J* = 5.6 Hz, 2H), 3.57 (s, 6H), 3.56 (s, 3H),

3.19 – 3.09 (m, 1H), 3.09 – 3.01 (m, 1H), 2.65 – 2.55 (m, 1H), 2.46 (s, 3H), 2.44 – 2.29 (m, 1H), 2.23 (dt, *J* = 14.5, 7.5 Hz, 1H), 2.16 (d, *J* = 13.2 Hz, 1H), 2.13 – 2.05 (m, 1H), 2.01 (dd, *J* = 12.4, 8.1 Hz, 1H), 1.97 – 1.86 (m, 1H), 1.79 (ddd, *J* = 18.5, 9.4, 5.1 Hz, 1H), 1.60 (qd, *J* = 15.0, 14.5, 9.6 Hz, 2H), 1.49 – 1.40 (m, 1H), 1.38 (d, *J* = 7.0 Hz, 3H), 1.33 (d, *J* = 6.9 Hz, 1H), 1.24 (s, 1H), 1.20 (d, *J* = 17.1 Hz, 6H), 0.94 (s, 9H), 0.81 (t, *J* = 7.3 Hz, 3H).

^{13}C NMR (126 MHz, DMSO) δ 172.47, 171.09, 171.00, 170.08, 167.69, 154.51, 153.14, 151.99, 149.07, 148.17, 147.48, 145.15, 136.67, 136.47, 136.02, 133.74, 131.62, 130.13, 129.29, 129.18, 126.85, 126.59, 121.59, 120.43, 112.62, 112.27, 105.53, 105.29, 70.15, 69.23, 67.56, 66.83, 60.48, 60.24, 59.01, 56.79, 56.71, 56.38, 55.98, 55.95, 55.76, 51.87, 49.13, 48.17, 43.36, 38.78, 38.19, 36.74, 35.66, 35.35, 31.02, 29.41, 28.90, 28.53, 27.14, 26.89, 26.81, 26.57, 25.85, 25.44, 22.89, 20.98, 16.42, 12.82, 12.73.

HRMS (ESI$^+$): *m/z* 1247.6302. Expected mass from chemical formula $C_{68}H_{90}N_6O_{14}S$: 1247.6308 Da.

LC/MS (ESI$^+$): *m/z* 1248 [M+H$^+$], 624 [M+H$^+$]/2.

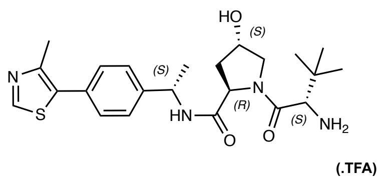

Supplementary Figure 12 | (2*R*,4*S*)-1-((*S*)-2-amino-3,3-dimethylbutanoyl)-4-hydroxy-*N*-((*S*)-1-(4-(4-methylthiazol-5-yl)phenyl)ethyl)pyrrolidine-2-carboxamide (6)

The title compound was prepared with minor modifications to the protocol previously described[1]. Briefly, trifluoroacetic acid (4N in DCM) was used in place of hydrochloric acid (4N in MeOH) to afford the title compound in quantitative yield.

^{1}H NMR (500 MHz, DMSO-d_6) δ 9.00 (s, 1H), 8.42 (d, *J* = 8.0 Hz, 1H), 8.09 (s, 2H), 7.48 – 7.44 (m, 4H), 4.95 (h, *J* = 7.1 Hz, 1H), 4.43 (ddd, *J* = 15.8, 8.5, 5.4 Hz, 2H), 3.92 (q, *J* = 5.5 Hz, 1H), 3.74 (dd, *J* = 10.9, 4.8 Hz, 1H), 3.58 (dd, *J* = 10.8, 3.3 Hz, 1H), 2.47 (s, 3H), 2.10 (ddd, *J* = 12.9, 8.3, 4.4 Hz, 1H), 2.01 – 1.94 (m, 1H), 1.38 (d, *J* = 7.0 Hz, 3H), 1.03 (s, 9H).
LC/MS (ESI$^+$): *m/z* 445[M+H$^+$]

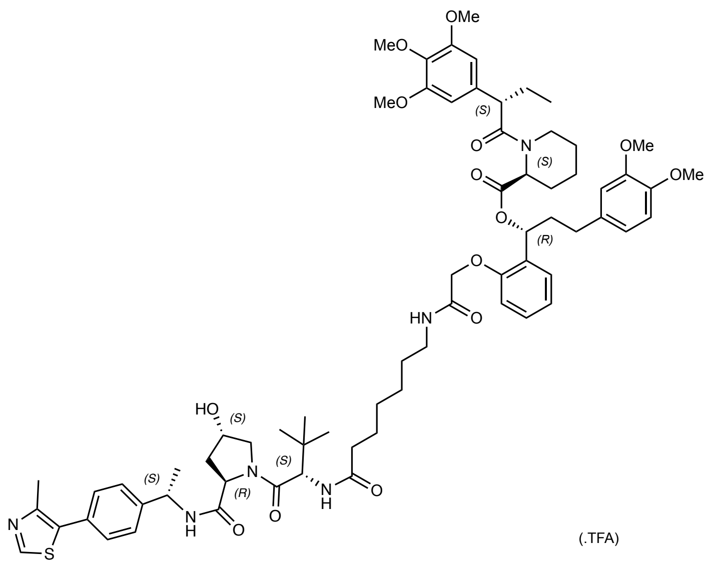

Supplementary Figure 13 | (*R*)-3-(3,4-dimethoxyphenyl)-1-(2-(2-((7-(((*S*)-1-((2*R*,4*S*)-4-hydroxy-2-(((*S*)-1-(4-(4-methylthiazol-5-yl)phenyl)ethyl)carbamoyl)pyrrolidin-1-yl)-3,3-dimethyl-1-oxobutan-2-yl)amino)-7-oxoheptyl)amino)-2-oxoethoxy)phenyl)propyl (*S*)-1-((*S*)-2-(3,4,5-trimethoxyphenyl)butanoyl)piperidine-2-carboxylate (dTAG^{V}-1-NEG)

The title compound was prepared from **5** according to scheme 1.

^{1}H NMR (500 MHz, DMSO-d_6) δ 8.98 (s, 1H), 8.03 (d, *J* = 8.0 Hz, 1H), 7.89 (d, *J* = 7.8 Hz, 1H), 7.68 (t, *J* = 5.8 Hz, 1H), 7.19 (ddd, *J* = 8.7, 6.6, 2.6 Hz, 1H), 6.86 (d, *J* = 8.3 Hz, 1H), 6.85 – 6.76 (m, 4H), 6.74 (d, *J* = 2.0 Hz, 1H), 6.65 – 6.60 (m, 2H), 6.56 (d, *J* = 2.7 Hz, 2H), 6.03 (dt, *J* = 8.5, 4.6 Hz, 1H), 5.32 (dd, *J* = 5.9, 2.5 Hz, 1H), 4.91 (h, *J* = 7.2, 6.5 Hz, 1H), 4.59 – 4.42 (m, 3H), 4.39 (dd, *J* = 8.0, 5.1 Hz, 2H), 4.31 (p, *J* = 5.2 Hz, 1H), 4.05 (d, *J* = 13.2 Hz, 1H), 3.86 (t, *J* = 7.2 Hz, 1H), 3.81 (dd, *J* = 10.5, 5.4 Hz, 1H), 3.74 (s, 2H), 3.71 (s, 3H), 3.69 (s, 3H), 3.64 (s, 1H), 3.56 (s, 6H), 3.55 (s, 3H), 3.50 (dd, *J* = 10.4, 4.2 Hz, 1H), 3.14 – 3.00 (m, 3H), 2.66 – 2.55 (m, 1H), 2.45 (d, *J* = 1.5 Hz, 3H), 2.43 – 2.30 (m, 1H), 2.24 (dt, *J* = 14.8, 7.7 Hz, 1H), 2.21 – 2.10 (m, 1H), 2.04 (ddd, *J* = 14.5, 8.1, 5.3 Hz, 1H), 2.00 – 1.85 (m, 3H), 1.67 – 1.48 (m, 3H), 1.47 – 1.32 (m, 2H), 1.31 (d, *J* = 7.0 Hz, 3H), 1.27 – 1.00 (m, 7H), 0.97 (s, 9H), 0.81 (t, *J* = 7.3 Hz, 3H).

^{13}C NMR (126 MHz, DMSO) δ 173.40, 172.45, 171.07, 171.01, 170.16, 167.70, 154.48, 153.13, 151.94, 149.05, 148.16, 147.46, 144.91, 136.45, 136.01, 133.71, 131.61, 130.09, 129.25, 129.15, 127.01, 126.60, 121.61, 120.43, 112.59, 112.33, 112.24, 105.52, 105.27, 70.10, 68.85, 67.52, 60.48, 60.23, 59.16, 57.54, 56.37, 55.97, 55.93, 55.74, 55.66, 51.86, 49.10, 47.99, 43.36, 38.74, 38.24, 36.70, 35.24, 34.71, 31.00, 29.34, 28.84, 28.50, 26.90, 26.76, 26.52, 25.76, 25.42, 22.96, 20.95, 16.40, 12.80, 12.70.

HRMS (ESI$^+$): *m/z* 1247.6288. Expected mass from chemical formula $C_{68}H_{90}N_6O_{14}S$:

1247.6308 Da.

LC/MS (ESI$^+$): *m/z* 1248[M+H$^+$], 624[M+H$^+$]/2.

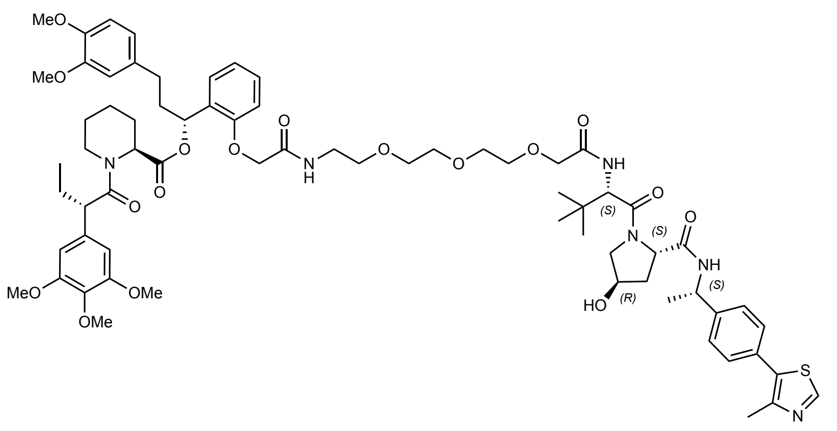

Supplementary Figure 14 | (*R*)-3-(3,4-dimethoxyphenyl)-1-(2-(((*S*)-16-((2*S*,4*R*)-4-hydroxy-2-(((*S*)-1-(4-(4-methylthiazol-5-yl)phenyl)ethyl)carbamoyl)pyrrolidine-1-carbonyl)-17,17-dimethyl-2,14-dioxo-6,9,12-trioxa-3,15-diazaoctadecyl)oxy)phenyl)propyl (*S*)-1-((*S*)-2-(3,4,5-trimethoxyphenyl)butanoyl)piperidine-2-carboxylate (dTAG-63)

The title compound was prepared from **1** according to Scheme 1.

^{1}H NMR (500 MHz, 1:1 CDCl3:Methanol-d4) δ 8.73 (s, 1H), 7.41 – 7.34 (m, 4H), 7.24 – 7.16 (m, 1H), 6.86 (t, J = 7.5 Hz, 1H), 6.79 (dd, J = 8.0, 6.0 Hz, 2H), 6.74 – 6.63 (m, 3H), 6.51 (s, 2H), 6.13 (dd, J = 7.8, 6.0 Hz, 1H), 5.44 (d, J = 4.8 Hz, 1H), 5.00 (q, J = 6.9 Hz, 1H), 4.61 (d, J = 6.6 Hz, 1H), 4.59 – 4.38 (m, 4H), 4.03 – 3.92 (m, 3H), 3.86 – 3.75 (m, 9H), 3.74 – 3.33 (m, 23H), 2.56 – 2.49 (m, 1H), 2.47 (s, 3H), 2.45 – 2.39 (m, 1H), 2.25 (d, J = 14.1 Hz, 1H), 2.16 (q, J = 8.0 Hz, 1H), 2.09 – 1.88 (m, 4H), 1.79 – 1.52 (m, 4H), 1.48 (d, J = 7.0 Hz, 3H), 1.21 (dd, J = 12.8, 3.5 Hz, 1H), 1.02 (s, 9H), 0.86 (q, J = 6.5, 5.8 Hz, 3H).

^{13}C NMR (126 MHz, 1:1 CDCl3:Methanol-d4) δ 173.75, 172.04, 171.75, 171.07, 170.96, 169.87, 154.61, 154.12, 153.87, 151.80, 149.46, 148.43, 147.94, 144.64, 137.33, 136.06, 134.33, 132.79, 130.86, 130.03, 129.98, 129.22, 127.66, 126.99, 126.85, 122.77, 121.10, 121.02, 112.67, 112.35, 112.17, 105.80, 105.19, 71.69, 71.13, 70.96, 70.75, 70.70, 70.59, 70.29, 69.78,

67.88, 61.08, 59.82, 57.48, 56.60, 56.40, 56.33, 56.26, 52.76, 50.87, 44.41, 39.47, 39.42, 37.80, 37.28, 36.59, 31.75, 30.19, 28.80, 27.14, 26.77, 25.89, 22.36, 21.40, 15.87, 12.59.

HRMS (ESI^+): *m/z* 1309.6301. Expected mass from chemical formula $C_{69}H_{92}N_6O_{17}S$: 1309.6312 Da.

LC/MS (ESI^+): *m/z* 1310 [$M+H^+$], 655 [$M+H^+$]/2.

Synthesis of dTAG-13-NEG

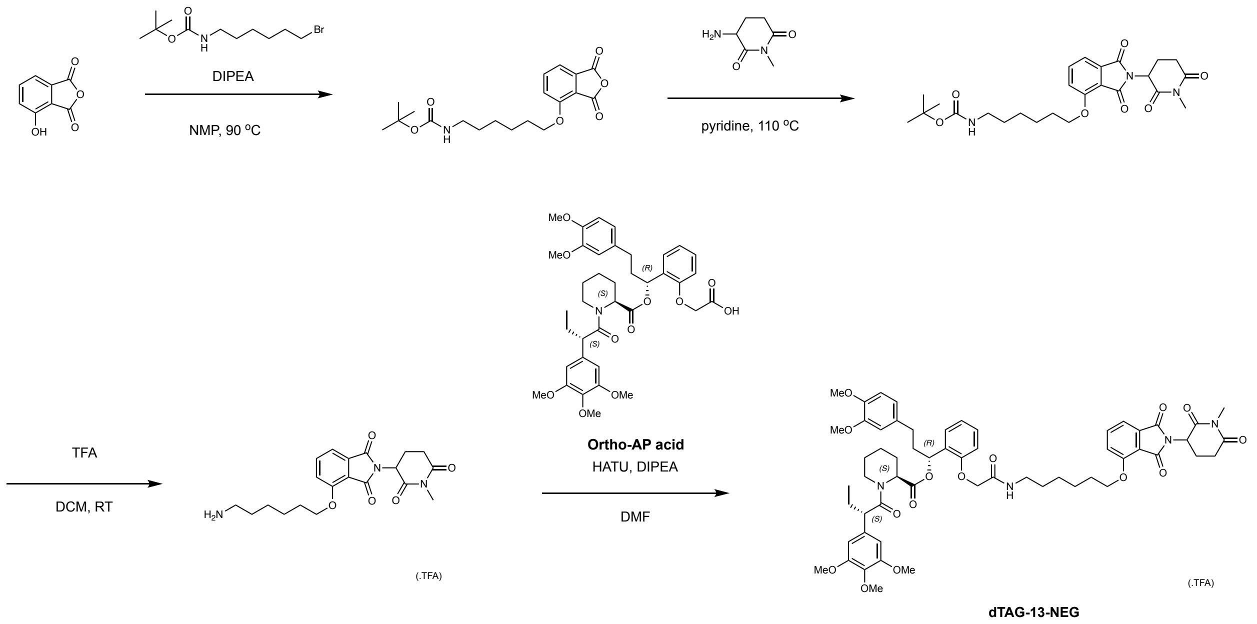

Supporting Figure 15 | Scheme used for synthesis of dTAG-13-NEG (Scheme 2).

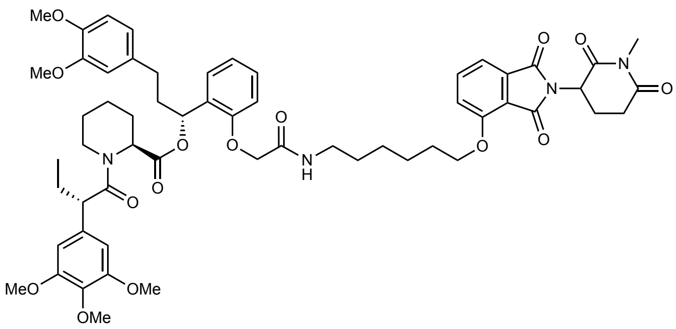

Supplementary Figure 16 | (1*R*)-3-(3,4-dimethoxyphenyl)-1-(2-(2-((6-((2-(1-methyl-2,6-dioxopiperidin-3-yl)-1,3-dioxoisoindolin-4-yl)oxy)hexyl)amino)-2-oxoethoxy)phenyl)propyl (2*S*)-1-((*S*)-2-(3,4,5-trimethoxyphenyl)butanoyl)piperidine-2-carboxylate (dTAG-13-NEG)

The title compound was prepared according to scheme 2, analogous to Nabet *et al.* and Erb *et al.*[2, 3]

^{1}H NMR (500 MHz, DMSO-d_6) δ 7.81 (t, *J* = 7.9 Hz, 1H), 7.69 (t, *J* = 5.8 Hz, 1H), 7.50 (dd, *J* = 8.6, 2.2 Hz, 1H), 7.44 (d, *J* = 7.2 Hz, 1H), 7.21 (ddd, *J* = 8.8, 5.8, 3.4 Hz, 1H), 6.88 (d, *J* = 8.3 Hz, 1H), 6.85 – 6.79 (m, 3H), 6.74 (d, *J* = 2.0 Hz, 1H), 6.66 – 6.61 (m, 2H), 6.56 (s, 2H), 6.04 (dd, *J* = 8.3, 4.9 Hz, 1H), 5.36 – 5.30 (m, 1H), 5.15 (dt, *J* = 13.1, 4.4 Hz, 1H), 4.49 (q, *J* = 14.6 Hz, 2H), 4.17 (q, *J* = 6.7 Hz, 2H), 4.05 (d, *J* = 13.3 Hz, 1H), 3.86 (t, *J* = 7.2 Hz, 1H), 3.74 (s, 2H), 3.71 (d, *J* = 1.9 Hz, 5H), 3.69 (s, 3H), 3.64 (s, 1H), 3.57 (s, 6H), 3.55 (s, 3H), 3.08 (q, *J* = 6.7 Hz, 1H), 3.01 (d, *J* = 2.2 Hz, 3H), 2.99 – 2.88 (m, 1H), 2.80 – 2.72 (m, 1H), 2.66 – 2.53 (m, 1H), 2.45 – 2.30 (m, 1H), 2.16 (t, *J* = 8.4 Hz, 2H), 2.08 – 1.85 (m, 3H), 1.70 (h, *J* = 6.2 Hz, 2H), 1.66 – 1.49 (m, 2H), 1.38 (p, *J* = 7.2, 6.7 Hz, 4H), 1.29 – 1.20 (m, 2H), 1.19 – 1.07 (m, 1H), 0.80 (t, *J* = 7.3 Hz, 3H).

^{13}C NMR (126 MHz, DMSO) δ 172.42, 172.24, 171.01, 170.16, 167.69, 167.30, 165.77, 156.49, 154.49, 153.30, 153.14, 149.06, 147.47, 137.52, 136.67, 136.45, 136.03, 133.72, 133.70,

129.18, 126.62, 121.61, 120.42, 120.21, 116.65, 115.62, 112.60, 112.37, 112.23, 105.52, 105.29, 70.11, 69.19, 67.58, 60.48, 60.23, 56.38, 55.97, 55.93, 55.75, 51.87, 49.77, 49.13, 49.02, 43.35, 40.90, 38.73, 36.72, 31.57, 31.02, 29.40, 28.82, 28.52, 28.18, 27.05, 26.76, 26.46, 25.49, 25.42, 21.67, 20.98, 18.55, 17.20, 12.81, 12.72.

HRMS (ESI^+): *m/z* 1063.4901. Expected mass from chemical formula $C_{58}H_{70}N_4O_{15}$: 1063.491 Da.

LC/MS (ESI) *m/z* 1064 $(M + H)^+$.

Synthesis of dTAG-47-NEG

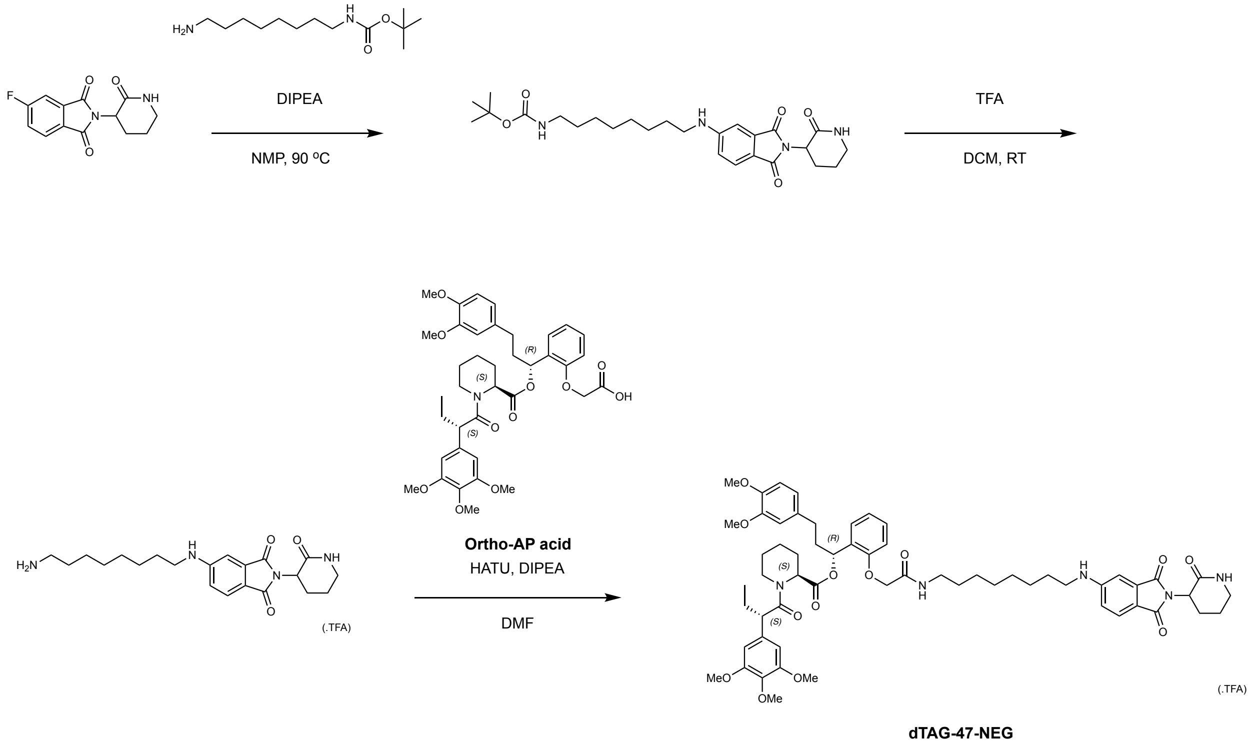

Supporting Figure 17 | Scheme used for synthesis of dTAG-47-NEG (Scheme 3).

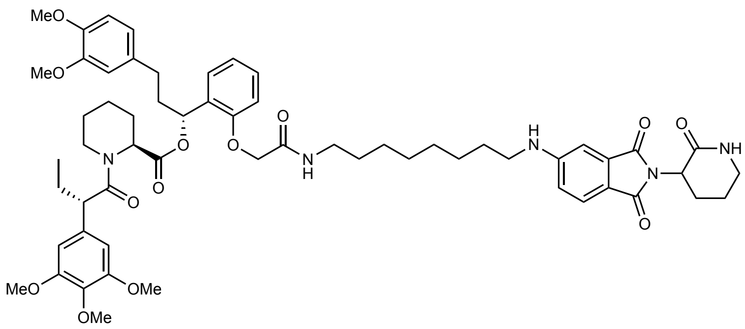

Supplementary Figure 18 | (1*R*)-3-(3,4-dimethoxyphenyl)-1-(2-(2-((8-((1,3-dioxo-2-(2-oxopiperidin-3-yl)isoindolin-5-yl)amino)octyl)amino)-2-oxoethoxy)phenyl)propyl (2*S*)-1-((*S*)-2-(3,4,5-trimethoxyphenyl)butanoyl)piperidine-2-carboxylate (dTAG-47-NEG)

The title compound was prepared according to scheme 3, analogous to Huang *et al.*[4]

^{1}H NMR (500 MHz, Methanol-d_4) δ 7.54 (d, J = 8.4 Hz, 1H), 7.25 (td, J = 7.8, 1.7 Hz, 1H), 6.96 (d, J = 2.2 Hz, 1H), 6.91 (t, J = 8.1 Hz, 2H), 6.84 (d, J = 8.2 Hz, 1H), 6.80 (ddt, J = 8.8, 7.4, 1.8 Hz, 2H), 6.76 (d, J = 2.0 Hz, 1H), 6.67 (dd, J = 8.2, 2.0 Hz, 1H), 6.64 (s, 2H), 6.14 (dd, J = 8.2, 6.0 Hz, 1H), 5.42 (d, J = 5.4 Hz, 1H), 4.69 (dd, J = 11.8, 6.0 Hz, 1H), 4.59 (d, J = 15.0 Hz, 1H), 4.44 (d, J = 15.0 Hz, 1H), 4.14 (d, J = 13.7 Hz, 1H), 3.88 (t, J = 7.3 Hz, 1H), 3.82 (d, J = 4.7 Hz, 6H), 3.80 (s, 3H), 3.77 (d, J = 6.6 Hz, 1H), 3.72 (s, 3H), 3.71 – 3.68 (m, 5H), 3.44 (td, J = 11.8, 3.8 Hz, 1H), 3.39 – 3.34 (m, 2H), 3.21 – 3.14 (m, 4H), 2.70 – 2.58 (m, 1H), 2.58 – 2.42 (m, 2H), 2.40 – 2.22 (m, 2H), 2.10 – 2.00 (m, 4H), 1.95 (dt, J = 13.9, 6.2 Hz, 1H), 1.74 (dt, J = 13.8, 7.0 Hz, 1H), 1.63 (p, J = 7.1 Hz, 2H), 1.59 – 1.45 (m, 1H), 1.44 – 1.36 (m, 4H), 1.35 – 0.99 (m, 6H), 0.89 (t, J = 7.3 Hz, 3H).

^{13}C NMR (126 MHz, DMSO) δ 172.44, 170.99, 168.38, 167.95, 167.77, 167.67, 154.76, 154.49, 153.31, 153.14, 149.08, 147.48, 136.46, 136.03, 134.82, 133.73, 129.18, 126.62, 125.31, 121.61, 120.42, 116.61, 112.61, 112.37, 112.24, 105.52, 105.29, 70.15, 67.59, 60.23, 56.38,

55.97, 55.94, 55.79, 55.76, 54.05, 51.86, 49.13, 46.21, 43.34, 42.95, 41.87, 40.90, 38.72, 36.74, 31.03, 29.45, 29.22, 28.71, 28.53, 26.99, 26.74, 26.43, 25.43, 22.26, 20.97, 18.55, 17.20, 12.93, 12.81, 12.73, 9.09.

HRMS (ESI^+): *m/z* 1062.5431. Expected mass from chemical formula $C_{59}H_{75}N_5O_{13}$: 1062.5434 Da.

LC/MS (ESI+) *m/z* 1063 $(M + H)^+$.

SUPPLEMENTARY REFERENCES

1. Raina, K. et al. PROTAC-induced BET protein degradation as a therapy for castration-resistant prostate cancer. *Proc. Natl. Acad. Sci. U. S. A.* **113**, 7124-7129 (2016).
2. Nabet, B. et al. The dTAG system for immediate and target-specific protein degradation. *Nat Chem Biol* **14**, 431-441 (2018).
3. Erb, M.A. et al. Transcription control by the ENL YEATS domain in acute leukaemia. *Nature* **543**, 270-274 (2017).
4. Huang, H.T. et al. MELK is not necessary for the proliferation of basal-like breast cancer cells. *Elife* **6**, e26693 (2017).
5. Olson, C.M. et al. Pharmacological perturbation of CDK9 using selective CDK9 inhibition or degradation. *Nat. Chem. Biol.* **14**, 163-170 (2018).
6. Winter, G.E. et al. BET Bromodomain Proteins Function as Master Transcription Elongation Factors Independent of CDK9 Recruitment. *Mol. Cell* **67**, 5-18 e19 (2017).
