# Supplementary material for: Rapid and direct control of target protein levels with VHL-recruiting dTAG molecules
Source: Nat Commun. 2020 Sep 18;11:4687. doi: 10.1038/s41467-020-18377-w (PMC7501296; doi:10.1038/s41467-020-18377-w)
Supplement: Supplementary file 2 — Description of Additional Supplementary Files [file 41467_2020_18377_MOESM2_ESM.pdf]

DESCRIPTION OF ADDITIONAL SUPPLEMENTARY FILES

File name: Supplementary Data 1
Description: Mass spectrometry-based proteomics raw data files.

File name: Supplementary Data 2
Description: Mass spectrometry-based proteomics processed data files.
