# Supplementary material for: Rapid and direct control of target protein levels with VHL-recruiting dTAG molecules
Source: Nat Commun. 2020 Sep 18;11:4687. doi: 10.1038/s41467-020-18377-w (PMC7501296; doi:10.1038/s41467-020-18377-w)
Supplement: Supplementary file 5 — Reporting Summary [file 41467_2020_18377_MOESM5_ESM.pdf]

nature research

Corresponding author(s): Behnam Nabet and Nathanael S. Gray

Last updated by author(s): July 23, 2020

Reporting Summary

Nature Research wishes to improve the reproducibility of the work that we publish. This form provides structure for consistency and transparency in reporting. For further information on Nature Research policies, see Authors & Referees and the Editorial Policy Checklist .

Statistics

For all statistical analyses, confirm that the following items are present in the figure legend, table legend, main text, or Methods section.

n/a	Confirmed	
☐	☒	The exact sample size (*n*) for each experimental group/condition, given as a discrete number and unit of measurement
☐	☒	A statement on whether measurements were taken from distinct samples or whether the same sample was measured repeatedly
☐	☒	The statistical test(s) used AND whether they are one- or two-sided<br>*Only common tests should be described solely by name; describe more complex techniques in the Methods section.*
☒	☐	A description of all covariates tested
☐	☒	A description of any assumptions or corrections, such as tests of normality and adjustment for multiple comparisons
☐	☒	A full description of the statistical parameters including central tendency (e.g. means) or other basic estimates (e.g. regression coefficient) AND variation (e.g. standard deviation) or associated estimates of uncertainty (e.g. confidence intervals)
☐	☒	For null hypothesis testing, the test statistic (e.g. *F*, *t*, *r*) with confidence intervals, effect sizes, degrees of freedom and *P* value noted<br>*Give P values as exact values whenever suitable.*
☒	☐	For Bayesian analysis, information on the choice of priors and Markov chain Monte Carlo settings
☒	☐	For hierarchical and complex designs, identification of the appropriate level for tests and full reporting of outcomes
☒	☐	Estimates of effect sizes (e.g. Cohen's *d*, Pearson's *r*), indicating how they were calculated

Our web collection on statistics for biologists contains articles on many of the points above.

Software and code

Policy information about availability of computer code

Data collection	Mass spectrometry: Orbitrap Fusion/Lumos (Thermo Fisher Scientific).
Data analysis	Mass spectrometry: Raw files were converted to mzXML using Raw File Reader (v3.0.77) provided by Thermo Fisher Scientific. Spectra were searched using Comet (2019.01.5). Search results were filtered using the LDA function in MASS Package in R as previously described (ref. 44). Spectral searches were performed using a 2020 Uniprot Human database.

For manuscripts utilizing custom algorithms or software that are central to the research but not yet described in published literature, software must be made available to editors/reviewers. We strongly encourage code deposition in a community repository (e.g. GitHub). See the Nature Research guidelines for submitting code & software for further information.

Data

Policy information about availability of data

All manuscripts must include a data availability statement. This statement should provide the following information, where applicable:

- Accession codes, unique identifiers, or web links for publicly available datasets
- A list of figures that have associated raw data
- A description of any restrictions on data availability

Mass spectrometry-based proteomics raw data files are provided in Supplementary Data 1 and have been deposited to the ProteomeXchange Consortium via the PRIDE partner repository (PXD018937). Mass spectrometry-based proteomics processed data files underlying Figs. 1d and 3c and Supplementary Fig. 1f are provided in Supplementary Data 2. The source data underlying Figs. 1c, 1e, 2a, 2b, 2c, 2d, 3a, 3b, 3d, 3f and Supplementary Figs. 1c, 1d, 1e, 2a, 2b, 2c, 2d, 2e, 4a, 4b, 4c, 4d, 5b, 5c, 5d, 6a, 6b, 6c are provided as a Source Data file.

Field-specific reporting

Please select the one below that is the best fit for your research. If you are not sure, read the appropriate sections before making your selection.

☒ Life sciences ☐ Behavioural & social sciences ☐ Ecological, evolutionary & environmental sciences

For a reference copy of the document with all sections, see nature.com/documents/nr-reporting-summary-flat.pdf

Life sciences study design

All studies must disclose on these points even when the disclosure is negative.

Sample size	Sample sizes were not predetermined using statistical analyses. Sample sizes were selected based on previous experience for each experiment (refs. 6 and 15). Information on the number of replicates and independent experiments that were performed for each measurement are disclosed in the manuscript.
Data exclusions	Data were not excluded from analysis.
Replication	Information on the number of replicates, samples and independent experiments that were performed for each measurement are disclosed in the manuscript. All biological experiments were successfully reproduced in n = 2 or n = 3 independent experiments. Quantitative proteomics experiments were performed using n = 2 or n = 3 independent biologically independent samples. Pharmacokinetic studies were performed using n = 3 biologically independent mice. Pharmacodynamic studies were performed using n = 4 or n = 5 biologically independent mice.
Randomization	Experiments were not randomized. Randomization was not applicable to this study as cell lines and animals were treated and assessed in the same manner with the appropriate controls.
Blinding	Experiments were not blinded. Blinding was not applicable to this study as data collection or analysis were not prone to bias. All experiments were precise as well as quantitative when possible and were not based on subject assessments.

Reporting for specific materials, systems and methods

We require information from authors about some types of materials, experimental systems and methods used in many studies. Here, indicate whether each material, system or method listed is relevant to your study. If you are not sure if a list item applies to your research, read the appropriate section before selecting a response.

Materials & experimental systems

n/a	Involved in the study	
☐	☒	Antibodies
☐	☒	Eukaryotic cell lines
☒	☐	Palaeontology
☐	☒	Animals and other organisms
☒	☐	Human research participants
☒	☐	Clinical data

Methods

n/a	Involved in the study	
☒	☐	ChIP-seq
☒	☐	Flow cytometry
☒	☐	MRI-based neuroimaging

Antibodies

Antibodies used	The following primary antibodies were employed in this study: HA (Cell Signaling, #3724 and #2367), phospho-ERK1/2 T202/Y204 (Cell Signaling, #4370), ERK1/2 (Cell Signaling, #4696), phospho-AKT S473 (Cell Signaling, #4060), AKT (Cell Signaling, #2920), FKBP12 (Abcam, #ab24373), GFP (Cell Signaling, #2555), FLI (Abcam, #ab15289), NKX2-2 (Abcam, #ab187375), GAPDH (Cell Signaling, #2118), Vinculin (Cell Signaling, #13901), beta-Actin (Cell Signaling, #58169), and alpha-Tubulin (Cell Signaling, #3873). Species-specific fluorescently labelled infrared secondary antibodies including IRDye 680LT anti-Mouse IgG (LI-COR #926-68020), IRDye 800CW anti-Mouse IgG (LI-COR #926-32210), IRDye 680LT anti-Rabbit IgG (LI-COR #926-68021), and IRDye 800CW anti-Rabbit (LI-COR #926-32211) and peroxidase-linked secondary antibodies including anti-Mouse IgG (Thermo Fisher Scientific #45000680) and anti-Rabbit IgG (Thermo Fisher Scientific #45000682) were employed as appropriate.
Validation	All antibodies employed in this study for immunoblotting are commercially available and were validated by the manufacturer as follows. For the HA (#3724 and #2367) antibodies from Cell Signaling Technology, the vendor shows detection of divergent, exogenously expressed HA-tagged fusions by immunoblotting in multiple cell lines. For the phospho-ERK1/2 T202/Y204 (#4370) antibody from Cell Signaling Technology, the vendor shows activation or suppression of phospho-ERK1/2 T202/Y204 in response to pathway activation (TPA treatment) or inhibition (U0126 or phosphatase treatment) by immunoblotting in multiple cell lines. For the ERK1/2 (#4696) antibody from Cell Signaling Technology, the vendor shows detection of ERK1/2 by immunoblotting in multiple cell lines. For the phospho-AKT S473 (#4060) antibody from Cell Signaling Technology, the vendor shows activation or suppression of phospho-AKT S473 in response to pathway activation (PDGF treatment) or inhibition (LY294002/wortmannin treatment) by immunoblotting in multiple cell lines. For the AKT (#2920) antibody from Cell Signaling Technology, the vendor shows detection of AKT by immunoblotting in multiple cell lines. For the FKBP12 (#ab24373) antibody from Abcam, the vendor

shows detection of FKBP12 and loss of FKBP12 upon knockout by immunoblotting. For the GFP (#2555) antibody from Cell Signaling Technology, the vendor shows detection of an exogenously expressed GFP-tagged fusion by immunoblotting. For the FLI (#ab15289) antibody from Abcam, the vendor shows detection of FLI by immunoblotting in multiple cell lines. For the NKX2-2 (#ab187375) antibody from Abcam, the vendor shows detection of FLI by immunohistochemistry in multiple tissues. For the GAPDH (#2118) antibody from Cell Signaling Technology, the vendor shows detection of GAPDH by immunoblotting in multiple cell lines. For the Vinculin (#13901) antibody from Cell Signaling Technology, the vendor shows detection of Vinculin by immunoblotting in multiple cell lines. For the beta-Actin (#58169) antibody from Cell Signaling Technology, the vendor shows detection of beta-Actin by immunoblotting in multiple cell lines. For the alpha-Tubulin (#3873) antibody from Cell Signaling Technology, the vendor shows detection of alpha-Tubulin by immunoblotting in multiple cell lines.

Eukaryotic cell lines

Policy information about cell lines

Cell line source(s)	The following cell lines were employed in this study: 293T (source: Thermo Fisher Scientific), 293FT (source: Thermo Fisher Scientific), PATU-8902 (source: DSMZ), MV4;11 (source: ATCC) and EWS502 (source: kindly provided by Dr. Stephen L. Lessnick of Nationwide Children's Hospital and established by Dr. Jonathan A. Fletcher of Harvard Medical School).
Authentication	293T, 293FT, PATU-8902 and MV4;11 cell lines were originally purchased directly from the vendor. Authentication was performed by the vendor using standard methods, which includes STR profiling. EWS502 cells were provided by Dr. Stephen Lessnick and STR profiling was performed at the Dana-Farber Cancer Institute.
Mycoplasma contamination	All cell lines tested negative for mycoplasma.
Commonly misidentified lines (See ICLAC register)	No commonly misidentified lines were used in this study.

Animals and other organisms

Policy information about studies involving animals; ARRIVE guidelines recommended for reporting animal research

Laboratory animals	8-week-old C57BL/6J male mice (Jackson Laboratory, #000664) and 8-week-old immunocompromised female mice (NOD.Cg-PrkdcscidIl2rgtm1Wjl/SzJ, NSG; Jackson Laboratory #005557) were employed in this study.
Wild animals	This study did not involve wild animals.
Field-collected samples	This study did not involve samples collected from the field.
Ethics oversight	All procedures were approved by and performed in accordance with standards of the Institute Animal Care and Use Committee (IACUC) at Scripps Florida and Dana-Farber Cancer Institute.

Note that full information on the approval of the study protocol must also be provided in the manuscript.
